# Recognition of eating episodes via commercial smartwatch sensors analysis

**DOI:** 10.1371/journal.pdig.0001539

**Published:** 2026-07-07

**Authors:** Luca Vedovelli, Mohammad Junayed Bhuyan, Corrado Lanera, Ileana Baldi, Paola Berchialla, Dario Gregori, NOTION Working Group

**Affiliations:** 1 Unit of Biostatistics, Epidemiology, and Public Health, Department of Cardiac, Thoracic, Vascular Sciences, and Public Health, University of Padova, Padova, Italy; 2 BIOSTAT-X Biostatistics & AI for Biomedical Discovery, Pediatric Research Institute “Città della Speranza”, Padova, Italy; 3 PhD Program in Translational Specialistic Medicine “G.B. Morgagni”, Curriculum “Biostatistics and Clinical Epidemiology”, University of Padova, Padova, Italy; 4 Department of Clinical and Biological Sciences, University of Torino, Torino, Italy; ETH Zurich, SWITZERLAND

## Abstract

Smartwatches with movement sensors have emerged as promising tools for monitoring dietary behavior. This study uses commercial smartwatch sensor data, namely tri-axial acceleration together with device-derived orientation (pitch and roll) and power features, to detect eating episodes, with rigorous subject-independent validation. Twenty healthy participants were equipped with smartwatches and video recorded during four meals under semi-naturalistic settings. Raw sensor data was transformed using time-windowed features. Multiple machine learning and deep learning methods were evaluated using Leave-One-Subject-Out (LOSO) cross-validation. Over 26,000 Information Units from 19 subjects were analyzed. Using only motion-derived predictors, XGBoost with hyperparameter tuning achieved the best balanced accuracy (0.639 [95% CI 0.607, 0.668]) and AUC (0.699 [0.653, 0.743]); the full feature set that additionally used experimental-context variables (meal, food and menu information) gave near-identical performance, and paired cluster-bootstrap comparisons indicated that Logistic Regression remained close on balanced accuracy. A Transformer encoder achieved the numerically highest sensitivity (0.691 [0.637, 0.735]) at a significantly lower specificity (0.504 [0.456, 0.549]); under nested threshold selection and multiplicity-adjusted comparison the sensitivity advantage over tuned XGBoost was not statistically significant. We demonstrate that eating detection from smartwatch sensors remains challenging when evaluated with proper subject-independent validation. The gap between within-subject and between-subject performance reflects high inter-individual variability in eating gestures, a key limitation for real-world deployment.

## Author summary

Diet-related diseases (obesity, diabetes, cardiovascular disease) kill more than fifty million people every year, and any effort to prevent them depends on knowing what and when people eat. Self-reported food diaries are known to be inaccurate, so the scientific community has been looking for a way to detect eating episodes automatically from the motion of a person’s wrist as captured by a smartwatch. Most published studies use research-grade motion sensors not available to the general public, and almost all evaluate their classifiers on the same individuals used to train them, which inflates performance estimates. We asked instead how well a *commercial* smartwatch (a Garmin Fenix 5, worn by twenty healthy adults during four standardised meals) can distinguish eating from non-eating using only its built-in motion signals, evaluated on *unseen* individuals. Our best classifier recognises the right eating movement roughly three times out of five; potentially useful for aggregate research, not yet good enough for clinical monitoring. We discuss several possible directions to close that gap, including a small amount of user-specific calibration data, larger and higher-frequency datasets, and fusion with additional sensors.

## 1. Introduction

Noncommunicable diseases (NCDs), including cardiovascular diseases, diabetes, and obesity, are responsible for 74% of global deaths annually, and are strongly linked to unhealthy eating behaviors [1]. Accurate and scalable dietary assessment is essential for maintaining overall human health and plays a crucial role in preventing and managing NCDs [2]. Wrist-worn devices, such as smartwatches, are particularly well-suited for this purpose as they can unobtrusively track characteristic arm and hand movements associated with eating activities, such as lifting utensils, sipping drinks, or bringing food to the mouth. These movements often display specific and repeatable patterns that can be identified using motion and orientation data collected from the sensors embedded in these devices [3].

Wearable technology, particularly smartwatches equipped with motion and orientation sensors, has opened new opportunities for tracking daily activities, including eating habits [4]. The automatic recognition of eating gestures and behaviors has become essential for behavioral research, nutritional management, and health monitoring [5,6]. Traditional dietary monitoring tools, such as food diaries or manual logs, are inherently error-prone, often compromised by memory lapses, estimation biases, and user non-compliance [7]. In contrast, automatic eating detection utilizing wearable sensor data offers a more dependable, continuous, and objective data collection without placing additional burdens on the user [8].

Despite this potential, several challenges remain. Detecting and isolating eating gestures from continuous motion data is particularly difficult. Human arm movements are highly variable and frequently include non-eating-related activities such as gesturing while speaking, scratching, or using a smartphone [9]. Eating gestures occur sporadically and are often interspersed with other unrelated movements, making it challenging to reliably extract relevant patterns from noisy datasets [3,9]. Furthermore, eating styles vary significantly across individuals and cultures. Different utensils (chopsticks, spoons, or hand-feeding) create diverse motion patterns that complicate algorithm standardization and require adaptable detection models [10,11].

Another critical limitation arises from the availability of labeled data [8]. Machine learning algorithms, which are widely used for gesture detection, rely on large, high-quality datasets for training and validation [12,13]. However, collecting sufficient data from free-living scenarios, where users go about their daily lives without external constraints, is both time-consuming and labor-intensive [14]. Moreover, datasets often suffer from class imbalance, with eating gestures significantly underrepresented. This limits model effectiveness and generalizability, highlighting the need for data augmentation techniques [15].

Over the past decade, significant progress has been made in addressing these challenges through advancements in machine learning and sensor-based analytics. Hidden Markov Models (HMMs), Convolutional Neural Networks (CNNs), and Support Vector Machines (SVMs) have all been successfully applied to detect eating gestures from smartwatch motion data [10,12,16]. Each of these approaches has its strengths: HMMs excel at capturing temporal dependencies in sequential data, while CNNs are highly effective at identifying spatial patterns in raw sensor signals [10,16]. Seven machine learning models - Support Vector Machine (SVM), Decision Tree (DT), Random Forest (RF), Logistic Regression (LR), Neural Networks (NN), Naïve Bayes (NB), and K-Nearest Neighbors (KNN) - were applied to classify eating gestures from motion sensor data [13]. Additionally, gesture segmentation techniques, such as wrist roll motion analysis and threshold-based peak detection, have further improved the accuracy of eating gesture recognition models [17].

This study focuses on the application of commercial smartwatch motion signals (tri-axial acceleration together with device-derived orientation and power features) for detecting eating episodes, aiming to provide non-intrusive, continuous monitoring of dietary behaviour in a free-living condition.

## 2. Methodology

### 2.1 Ethics statement

This study was conducted in accordance with the Declaration of Helsinki. The study protocol was approved by the Bioethics Committee of the University of Torino (Italy), acting as the Institutional Review Board, on 12/7/2017 (protocol number 256091) and renewed on 10/06/2024 (protocol number 399) by the Ethics Committee of the IRCCS Istituto Oncologico “Gabriella Serio”, Bari (Italy), ID: 1619/CEL. All participants were healthy adults aged between 20 and 30 years. Informed consent was obtained from every participant before enrolment, including explicit consent for the video recording and the analysis of the derived sensor data.

### 2.2 Study design and participants

**Fig 1:**
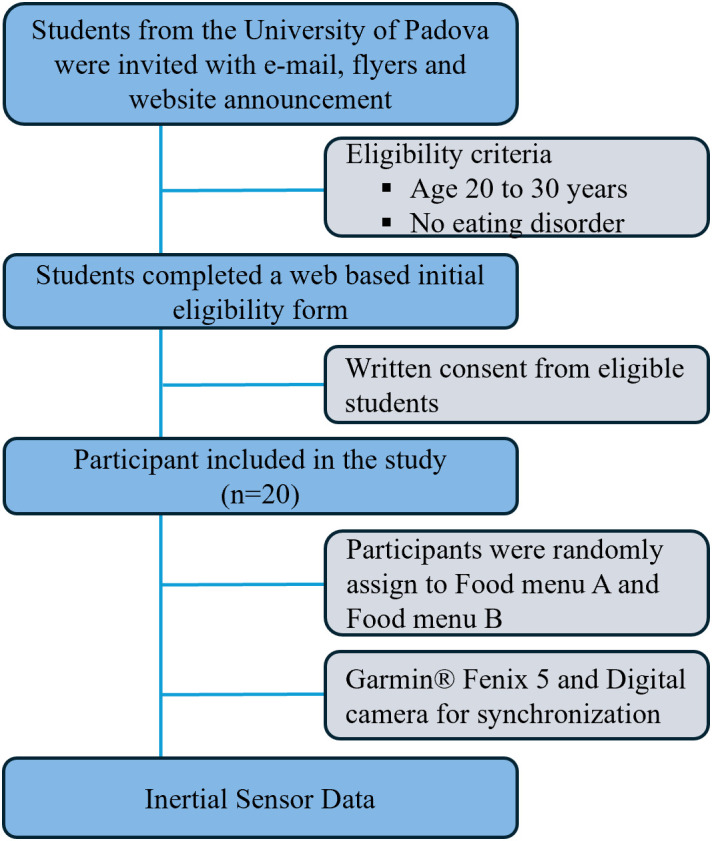
NOTION study design and participant flow. Flow chart of the NOTION (measuriNg calOric inTake at populatION level) project: recruitment of healthy adults aged 20–30 years, eligibility screening, randomisation to one of two standardised menus (Menu A or Menu B) across four eating occasions (breakfast, lunch, snack, dinner), video-recorded data collection in a semi-naturalistic setting (university cafeteria), synchronisation of wrist-worn Garmin Fenix 5 sensor streams with video footage, independent labelling of eating movements by two evaluators, and derivation of the eating_union (EU) outcome used for classification.

The study was conducted using data from NOTION project (measuriNg calOric inTake at populatION level), which investigated the use of wearable devices for dietary monitoring at a population level [18]. Individuals with food allergies, diagnosed eating disorders, or medical conditions likely to affect dietary behavior were excluded (Fig 1). Table 1 represents the characteristics of the study participants by sex.

**Table 1:** Characteristics of study participants- the values represent the mean (standard deviation) of characteristics of participants.

Characteristics	Women (n = 9)	Men (n = 11)	Total (n = 20)
Age (year)	23.3 (3.24)	23.4 (3.45)	23.4 (3.27)
Weight (kg)	59.8(5.22)	76.4 (8.96)	68.9 (11.2)
Height (cm)	166 (5.67)	181 (6.12)	174 (9.64)
BMI, (kg/m2)	21.7 (1.83)	23.2 (2.83)	22.5 (2.50)
Waist circumference (cm)	69.8 (4.29)	76.9 (5.60)	73.7 (6.12)
Hip circumference (cm)	93.0 (4.19)	94.6 (6.16)	93.9 (5.29)
Bicipital skinfold (mm)	7.64 (3.04)	5.25 (1.80)	6.33 (2.66)
Tricipital skinfold (mm)	18.1 (4.37)	11.9 (4.75)	14.7 (5.47)
Subscapular skinfold (mm)	15.2 (5.41)	14.6 (5.71)	14.8 (5.44)
Suprascapular skinfold (mm)	13.6 (3.82)	11.6 (3.56)	12.5 (3.72)

Participants were randomly assigned to consume predefined meals under semi-naturalistic conditions (i.e., no movement restrictions but with standardized meals). To ensure a free-living meal environment, the experimental phase was conducted in an authorized public catering area (a university cafeteria) frequented by students and staff. The meals were divided into four eating occasions, including breakfast, lunch, snack, and dinner. Each meal consisted of various food items, totaling thirteen distinct foods, which were evenly distributed across two predefined menus labeled as Menu A and Menu B. The consumption of these food items was video recorded for later synchronization with sensor data.

One participant (subject 02) was excluded from all subsequent analyses because they were left-handed: the feature set used for classification is extracted from the right wrist (Methods §2.3), which is the dominant wrist of the remaining 19 right-handed participants. With only one left-handed subject in the cohort, no formal test of the dominance effect was possible, so the conservative choice was to exclude this participant.

### 2.3 Devices and data collection

**Fig 2:**
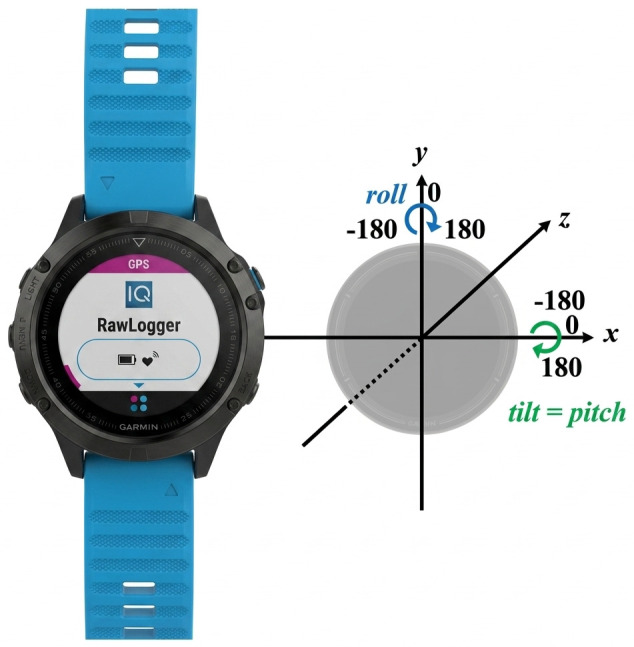
Kinetic variables captured by the Garmin Fenix 5 smartwatch. Schematic of the sensor axes used in the study. The device records tri-axial acceleration along the x, y and z axes (expressed in milli-g, where g denotes Earth’s gravitational acceleration), together with orientation metrics: roll (rotation about the y axis) and pitch (rotation about the x axis, referred to as tilt in the panel), both expressed in radians. Power is computed as the Euclidean norm of the 3D acceleration vector; total energy as the difference between the squared power and 1 g^2^. The photograph was taken by the corresponding author on the author’s own device; schematic overlays were drafted with the assistance of Google Gemini and subsequently reviewed and edited by the authors.

Two commercial Fenix 5 smartwatches (Garmin, Schaffhausen, Switzerland) were used to collect motion data from both wrists of each participant (Fig 2). These smartwatches provided tri-axial acceleration together with device-derived orientation (pitch and roll) and power features at a frequency of 5 Hz, capturing motion in real time. The devices measured kinetic variables including acceleration along the x, y, and z axes, pitch (tilt around the x-axis), roll (rotation around the y-axis), power (Euclidean norm of acceleration), and total energy (power purged of gravitational effects). Raw data were recorded using the RawLogger app for Garmin [19].

To synchronize the smartwatch data with video recordings, participants were instructed to perform a single hand clap before consuming each food item. This produced a distinct spike in both the motion data and the video’s audio channel, allowing for precise alignment during post-processing. Video recordings were captured at a frame rate of 25 FPS. Two independent raters, trained on a shared rubric, annotated the video recordings frame-by-frame to identify every eating movement. Following the NOTION labelling protocol [18], a raw sample was labelled ‘eating’ if, at that time point, food or beverage was being brought to, or was placed into, the mouth; the start of an eating movement was the video frame in which the food item (or cup) first left the plate on a trajectory toward the mouth, and the stop was the first frame after the food had entered the mouth and the hand had begun to retract. Movements adjacent to the eating gesture (cutlery manipulation, food mixing, cutting, wiping, talking, scratching, and use of a smartphone) were annotated as non-eating. Drinking gestures followed the same rubric, with the cup treated analogously to a food item. Annotations were recorded in an Excel template at the video frame rate, then projected onto the sensor stream using the hand-clap markers described above (§2.2) to produce two independent binary label streams per participant at the 5 Hz sensor rate (one stream per rater); these two streams were combined into the eating-union and eating-intersect labels used throughout, and were also compared directly, to quantify inter-rater agreement (§2.5.7; S1 Appendix Table S1.4 in S1 Appendix).

### 2.4 Sensors’ signals

Raw sensor data from the Fenix 5 devices were initially recorded in FIT format and converted to CSV using FitCSVTool.jar from the Garmin FIT-SDK [20]. These files were then processed in R [21] using the *tidyverse (v.2.0)* and *caret (v.6.0)* [22] packages. The six motion channels exposed by the RawLogger API and retained as kinetic variables were tri-axial acceleration (acc_x, acc_y, acc_z, in milli-g), orientation (pitch and roll, in radians, both derived by the device’s internal sensor-fusion algorithm from the underlying inertial measurement unit), and power (the Euclidean norm of the 3D acceleration vector). No raw gyroscope stream is directly exposed by the RawLogger API; the pitch and roll orientation variables are the device-published derivatives of the IMU output. Net total energy expenditure was derived from the difference between the square of power and 1 g² [23]. The final set of kinetic variables consisted of seven metrics: acc_x, acc_y, acc_z, pitch, roll, power, and total energy.

### 2.5 Datasets

The raw data from the devices were recorded at a rate of 5 Hz, meaning 5 data points were collected per second. We defined a window as any consecutive sequence of raw data within a fixed time duration. To analyze the data, we created windows of various time widths: 1, 2, 3, 4, and 5 seconds, corresponding to 5, 10, 15, 20, and 25 consecutive data points, respectively. For each time width, all possible overlapping windows were generated and summarized into a single Information Unit (IU) by considering the slopes (beta) of the regression line of the corresponding kinetic variables against time, estimated along the window itself. Windows advanced with a stride of 1 second (5 samples at 5 Hz) and were generated within each (subject, meal) recording session: they did not cross meal boundaries, although a window could span the food sub-segments within a meal. After exclusion of the single left-handed participant (subject 02), this produced 26,304 Information Units at the adopted window length of 5 seconds, computed from right-wrist data only. We also summarized the raw data into IU by mean, median, maximum, minimum, interquartile range (IQR), standard deviation (SD), and coefficient of variation (CV), corresponding to each kinetic variable within the time windows. These IUs were then labeled as ‘eating’ based on a specific criterion: an IU was assigned the label ‘eating’ if more than 50% of the raw data points within the window were individually labeled as ‘eating’; otherwise, it was labeled as ‘non-eating’. To select the window length δs for downstream analyses, we ran a sensitivity analysis under the same Leave-One-Subject-Out cross-validation used for the main results, fitting XGBoost with its default hyperparameters on each of the five candidate window sizes (δs ∈ {1, 2, 3, 4, 5} s). Cluster-bootstrap 95% confidence intervals for sensitivity, specificity, balanced accuracy and AUC overlapped across all five candidate lengths (S3 Table): the maximum difference in balanced accuracy across δs was 0.033, well within the width of any individual confidence interval. We adopted δs = 5 s for all subsequent analyses on three grounds: (i) at 5 Hz sampling, a 5-second window yields 25 raw samples per window statistic, stabilising the estimation of mean, SD, IQR and CV relative to the 5-sample support available for δs = 1 s; (ii) this window length is consistent with the typical duration of a complete intake gesture reported in the wrist-worn eating-detection literature [14,24]; (iii) as shown in S3 Table, the qualitative ranking of models and the directional conclusions of the paper are robust to the choice of δs within the tested range.

The full preprocessing pipeline, implemented in R with the *tidymodels* framework, removes the subject identifier (to prevent subject-identity leakage during training), dummy-encodes the three categorical covariates (arm, meal, food), removes zero-variance predictors, and z-score-normalises all numerical predictors. Applied to the δs = 5 s subset, this pipeline produces 75 numerical predictors that are fed to each machine-learning classifier in Table 2: 7 slope (linear-regression) coefficients of the raw signals against time; 49 window statistics computed on the right-wrist device (mean, median, minimum, maximum, inter-quartile range, standard deviation, and coefficient of variation of each of the 7 kinetic variables); 3 axis cross-correlations; and 16 categorical dummies (1 for arm, 3 for meal and 12 for food). The complete list of predictor names is reported in S4 Table and is also provided as a plain-text CSV in the companion repository. The time-series classifiers (Attention pooling + MLP and the Transformer encoder, Table 3) operate on the raw 25-timestep × 6-channel signal instead of the aggregated predictors and are described separately in §2.5.4 and S2 Table.

**Table 2:** Performance* of machine-learning models under LOSO cross-validation (δs = 5 s). Every metric is reported as “point estimate [95% CI]” with subject-level cluster-bootstrap intervals.

Model	Sensitivity	Specificity	Balanced accuracy	AUC
Logistic Regression	0.625 [0.547, 0.707]	0.643 [0.584, 0.705]	0.634 [0.609, 0.659]	0.697 [0.665, 0.728]
Decision Tree	0.572 [0.482, 0.653]	0.659 [0.595, 0.723]	0.616 [0.589, 0.641]	0.649 [0.615, 0.682]
Random Forest (default)	0.224 [0.153, 0.290]	0.931 [0.903, 0.958]	0.578 [0.554, 0.599]	0.700 [0.658, 0.741]
Random Forest (tuned)	0.240 [0.174, 0.311]	0.915 [0.883, 0.948]	0.578 [0.554, 0.601]	0.691 [0.648, 0.728]
XGBoost (default)	0.417 [0.341, 0.491]	0.814 [0.765, 0.858]	0.615 [0.589, 0.638]	0.693 [0.656, 0.726]
**XGBoost (tuned)**	0.593 [0.512, 0.677]	0.692 [0.624, 0.756]	**0.642 [0.612, 0.671]**	**0.712 [0.671, 0.749]**
LightGBM (default)	0.295 [0.221, 0.379]	0.884 [0.844, 0.921]	0.590 [0.565, 0.617]	0.691 [0.654, 0.729]
LightGBM (tuned)	0.281 [0.204, 0.369]	0.893 [0.852, 0.929]	0.587 [0.560, 0.614]	0.696 [0.652, 0.734]

*The performance reported as the headline in the text is based on the sensor-only model, which excludes the study-arm, meal, and food variables and uses the 59 motion-derived predictors: it gives balanced accuracy 0.639 and AUC 0.699, near-identical to the full-feature model shown here (S1 Appendix). The tuned estimates are post-selection leave-one-subject-out estimates; a fully nested subject-level cross-validation confirms them (S1 Appendix).

**Table 3:** Performance of time-series/ deep-learning models under LOSO cross-validation (δs = 5 s). Every metric is reported as “point estimate [95% CI]” with subject-level cluster-bootstrap intervals.

Model	Decision threshold	Sensitivity	Specificity	Balanced accuracy	AUC
Attention pooling (default)	0.50	0.521 [0.471, 0.574]	0.698 [0.660, 0.736]	0.609 [0.587, 0.632]	0.642 [0.613, 0.672]
Attention pooling (tuned)	0.50	0.577 [0.509, 0.652]	0.662 [0.611, 0.712]	0.620 [0.593, 0.644]	0.669 [0.634, 0.702]
Transformer*	0.30	0.691 [0.637, 0.735]	0.504 [0.456, 0.549]	0.597 [0.570, 0.624]	0.646 [0.613, 0.679]

*The Transformer decision threshold (θ = 0.30) was selected on pooled leave-one-subject-out predictions. Under fully nested threshold selection the Transformer sensitivity is 0.649, and its sensitivity advantage over tuned XGBoost is not statistically significant after multiplicity adjustment, while its specificity is significantly lower (S1 Appendix).

Each Information Unit (IU) was then assigned two summary labels derived from the two rater streams and from the same >50% majority-label rule applied to the raw samples in the window: the *Eating Union* (EU = 1 if at least one of the two raters labelled the window majority as ‘eating’) and the *Eating Intersect* (EI = 1 if both raters labelled the window majority as ‘eating’; EI = 1 ⇒ EU = 1 by construction). All main analyses reported below target the EU label, which is the more sensitivity-oriented of the two and better reflects the clinical priority of not missing genuine eating episodes; however, we also use EI as the target of a robustness analysis to bound the influence of label-boundary noise on the reported performance (§2.5.7 and §3.5).

### 2.6 Classification models and validation strategy

#### 2.6.1 Validation strategy.

To ensure realistic performance estimates generalizable to unseen individuals, we employed Leave-One-Subject-Out (LOSO) cross-validation. In each fold, data from 18 subjects were used for training, and the held-out subject served as the test set. This approach prevents subject-specific patterns from leaking into the model, providing conservative but realistic performance estimates [25]. Because the tuned-model hyperparameters and the Transformer decision threshold were selected on the same leave-one-subject-out folds used for reporting, the tuned estimates in Tables 2 and 3 are post-selection leave-one-subject-out estimates rather than fully independent final-test estimates. To confirm that this selection did not inflate the reported performance, we repeated the analysis with a fully nested subject-level cross-validation, in which all hyperparameters and the decision threshold were selected on the inner 18 subjects through a subject-grouped five-fold inner cross-validation and evaluated once on the held-out subject; the nested and post-selection estimates differed by at most 0.005 in balanced accuracy for the tuned tree models, with no evidence of post-selection optimism, and the full nested results are reported in S1 Appendix Table S1.1 in S1 Appendix.

#### 2.6.2 Class imbalance handling.

The dataset exhibited substantial class imbalance, with eating episodes representing approximately 26.9% of observations (§3.1). To address this, we applied class weights during model training: for tree-based models, we used *scale_pos_weight* (ratio of non-eating to eating samples); for neural network approaches, we employed weighted loss functions and oversampling of the minority class.

#### 2.6.3 Machine learning models.

Logistic Regression and Decision Tree were evaluated as naïve baselines under the same LOSO protocol used for the ensemble models (Table 2). We additionally evaluated three ensemble learning frameworks with established performance on tabular classification tasks. Random Forest [26] aggregates predictions from multiple decision trees trained on bootstrap samples, with class weights applied to address imbalance. XGBoost [27] employs gradient boosting with L1/L2 regularization, using the *scale_pos_weight* parameter to penalize misclassification of the minority eating class. LightGBM [28] implements gradient boosting with leaf-wise tree growth, offering native support for class weighting and computational efficiency on large datasets. Hyperparameter optimization followed a Latin Hypercube sampling strategy with racing-based early stopping to reduce computational cost [29]. The search space included the number of trees (100–1000), maximum tree depth (3–15), learning rate (0.001–0.3), and minimum samples per leaf (2–40). The configurations selected on LOSO balanced accuracy and used in Table 2 were: XGBoost tuned on the Eating Union (EU) target, *trees* = 599, *tree_depth* = 4, *learning_rate* = 0.0135, *min_n* = 13; LightGBM tuned, *trees* = 624, *tree_depth* = 10, *learning_rate* = 0.044, *min_n* = 8; Random Forest tuned, with *mtry* and *min_n* optimised over the same Latin-hypercube search and *trees* = 500 — the tuned configuration did not meaningfully change the operating point of the default Random Forest (balanced accuracy 0.578 vs 0.578, sensitivity 0.240 vs 0.224). The XGBoost configuration re-tuned on the Eating Intersect (EI) target (§3.5) was *trees* = 388, *tree_depth* = 5, *learning_rate* = 0.019, *min_n* = 5. Full search-space outputs and selected configurations are provided in the companion repository.

#### 2.6.4 Time-series classifiers.

To exploit the temporal structure of the raw sensor stream (25 timesteps × 6 channels per Information Unit: the six channels exposed directly by the RawLogger API, namely acc_x, acc_y, acc_z, pitch, roll, and power; the derived total-energy channel used by the machine-learning classifiers of Table 2 is a deterministic transformation of power and is omitted here to avoid feeding the time-series networks redundant information), we evaluated two complementary time-series classifiers. The first uses *deterministic* multi-head attention pooling as a feature extractor, followed by a compact multilayer perceptron; the second is an *end-to-end* learnable Transformer encoder. Complete architectural details, regularisation settings, and trainable-parameter counts are reported in S2 Table.

***Attention pooling with MLP classifier.*** Raw windows were summarised by four attention heads implementing deterministic, hand-crafted weighting schemes (energy-based, acc_x-magnitude-based, gradient-based, and uniform) each followed by softmax with temperature τ = 1.0 and a weighted sum over timesteps. These 4 pooled views, together with 26 auxiliary descriptors (weighted mean, per-channel variance, per-channel mean absolute gradient, attention entropy, and peak-attention timestep), produce a 50-dimensional representation. No attention parameters are learned: the attention weights of this model are fully defined by the input signal. The 50-dimensional feature vector is then fed to a single-hidden-layer MLP (50 → 64 → 2, L2 regularisation α = 10 ⁻ ³, learning rate 10 ⁻ ⁴, early stopping on a 10% validation split; scikit-learn). Balanced training was obtained by oversampling the minority (eating) class with replacement. The total number of trainable parameters is 3,394, corresponding to a parameter-to-sample ratio of ≈ 0.14: 1 across the ~ 25,000 IUs available at each LOSO fold.

***Transformer encoder.*** The Transformer branch is a learnable architecture implemented in PyTorch. An input linear projection maps the 6-channel signal to d_model = 64; a learnable positional embedding (25 × 64 parameters) is then added. Four stacked nn.TransformerEncoderLayer blocks (d_model = 64, 4 attention heads, feed-forward width = 128, dropout = 0.4) perform self-attention and position-wise feed-forward mixing over the 25-step sequence. Sequence representations are reduced by global average pooling and classified by a two-layer head (64 → 32 → 1) with dropout in between. The decision threshold for the sigmoid output was selected post hoc by balanced-accuracy grid search on LOSO predictions (θ = 0.30); all other classifiers retained the default decision threshold of 0.5. Training used AdamW (learning rate 5 × 10 ⁻ ⁴, weight decay 10 ⁻ ⁴) with a ReduceLROnPlateau scheduler (factor 0.5, patience 5), gradient clipping at max-norm 1.0, a WeightedRandomSampler for class balance in each minibatch, BCEWithLogitsLoss with pos_weight, and early stopping on the training-loss plateau (patience 15 epochs). The selected architecture has 138,049 trainable parameters, i.e., a parameter-to-sample ratio of ≈ 5.5: 1.

***Overfitting prevention.*** Given the moderate training-sample size (≈ 25,000 IUs from 18 subjects per LOSO fold) and the non-trivial parameter count of the Transformer, we adopted four complementary safeguards: (i) subject-independent evaluation by LOSO cross-validation, which is the primary and structural defence against the memorisation of subject-specific signatures, as no subject ever appears in both training and test; (ii) explicit capacity control (d_model ≤ 64, ≤ 4 encoder layers, feed-forward width ≤ 128) relative to the few-thousand sample regime; (iii) strong stochastic regularisation (dropout 0.4 on positional, attention, and feed-forward paths for the Transformer; α = 10 ⁻ ³ L2 regularisation for the MLP); and (iv) training-dynamics regularisation (AdamW weight decay 10 ⁻ ⁴, gradient clipping at max-norm 1.0, learning-rate reduction on plateau, and early stopping). None of the input features provided to either classifier identifies the subject, meaning any residual subject-specific pattern must be learned indirectly, and even then, it is tested only on unseen subjects.

#### 2.6.5 Evaluation metrics.

Given the class imbalance and the clinical importance of correctly identifying eating episodes, we report sensitivity (proportion of eating episodes correctly detected), specificity (proportion of non-eating periods correctly classified), balanced accuracy (arithmetic mean of sensitivity and specificity), and area under the receiver operating characteristic curve (AUC-ROC).

#### 2.6.6 Statistical analysis.

Evaluation metrics (sensitivity, specificity, balanced accuracy, AUC- ROC) are reported as means across the 19 LOSO subjects, together with 95% confidence intervals obtained by a subject-level (cluster) non-parametric bootstrap [30,31]. At each of B = 1,000 iterations we sampled 19 subjects with replacement from the 19 LOSO folds and recomputed the mean-across-subjects of each metric; the 2.5th and 97.5th percentiles of the resulting bootstrap distributions are the reported CI bounds. Because LOSO cross-validation treats each subject as the unit of independent replication, this subject-level resampling correctly propagates the between-subject variability into the uncertainty of the aggregated estimates; instance-level (per-Information-Unit) intervals, by ignoring clustering, would have systematically understated this uncertainty. All analyses were performed in R version 4.4 using the *yardstick* and *tidyverse* packages; the bootstrap script and per-subject metrics are provided in the companion GitHub repository (lucavd/recognition_of_eating_episodes).

#### 2.6.7 Labelling reliability and label-noise robustness.

Because the annotation boundary between eating and non-eating is inherently subjective (a one-second shift at 5 Hz translates into five raw samples and therefore ~20% of a 5-second window) we quantify inter-rater reliability at the IU level and use the EI target as a label-robust benchmark for the main result. At the IU level, agreement between raters is summarised by the percent agreement, computed as the proportion of IUs on which both raters agreed on the binary summary label (EI = 1 or EU = EI = 0), and by Cohen’s kappa, both computed directly from the two individual rater streams. We report agreement at the IU level and, as additional context, at the raw-frame (5 Hz) level; the rater-by-rater contingency tables are given in the S1 Appendix. Labelling reliability is reported overall (across the 19 subjects, 26,304 IUs with δs = 5 s) and per-subject (S1 Appendix Table S1.4 in S1 Appendix and the companion repository). As an additional robustness check, we refit the best-performing model (tuned XGBoost) with the EI target in place of EU, following the same Latin-hypercube tuning and LOSO evaluation protocol used for Table 2 (see §3.5).

#### 2.6.8 Few-shot personalisation.

To quantify the expected benefit of a lightweight user-level calibration step, we performed a controlled personalisation experiment on the best-performing classifier (tuned XGBoost), using the sensor-only feature set. To prevent the calibration and the evaluation Information Units from sharing raw signal, which the one-second stride would otherwise allow, calibration and testing were blocked by meal: for each subject we drew n ∈ {5, 10, 20, 50, 100} eating Information Units from that subject’s first two meals (or all available, when a subject’s first two meals contained fewer), fine-tuned the whole-cohort classifier on those subject-specific samples, and evaluated on that subject’s remaining two meals, with the baseline and the personalised models assessed on the same held-out meal block. Windows within a single meal still overlap, so this design removes the overlap between the calibration and the evaluation sets but not the temporal correlation among windows of the same meal. The experiment was run on all 19 LOSO subjects, with a separate pre-specified subset of five difficult subjects, identified as the bottom five participants by baseline LOSO sensitivity under XGBoost with default hyperparameters (subjects 08, 09, 10, 11, 16; baseline sensitivity range 0.16 to 0.29, mean 0.21), for which the expected personalisation benefit is largest.

## 3. Results

### 3.1 Dataset characteristics

Rather than applying a signal-level low-pass filter upstream of the feature extraction, we adopted a feature-level denoising strategy: each 5-second Information Unit is summarised by seven window statistics (mean, median, minimum, maximum, inter-quartile range, standard deviation and coefficient of variation of each of the seven kinetic variables) together with the slope of a linear regression of the raw signal against time, so that the classifier receives both the central tendency and the noise envelope of the window and learns their separation from data. Predictors are then z-score-normalised and zero-variance terms removed within the preprocessing recipe described in §2.4. This approach is consistent with the feature-first pipelines of Thomaz et al. [24] and Stankoski et al. [8], and distinct from the filter-first pipelines of Dong et al. [14] and Kyritsis et al. [32], which pair an explicit low-pass filter with IMUs sampling at 60–62 Hz. To validate this choice empirically, we compared our pipeline with and without a 2nd-order zero-phase Butterworth 0.3 Hz low-pass filter (the cut-off proposed in the original NOTION protocol [18]): the filter produced a statistically significant loss of sensitivity (Δ = −0.033, p = 0.002) without compensating gains in balanced accuracy or AUC (S6 Table), confirming that the window-level aggregation preserves more eating-relevant information than a hard low-pass stage on our 5 Hz data.

After preprocessing with a 5 s time window, the dataset contained 26,304 Information Units from the 19 subjects retained for LOSO cross-validation (subject 02, left-handed, was excluded; see Methods §2.1). The class distribution showed 26.9% eating episodes (7,080 IUs) and 73.1% non-eating episodes (19,224 IUs), yielding an overall class-imbalance ratio of 2.72: 1 (Non-eating: Eating). Per-subject fold sizes ranged from 814 to 1,930 IUs (mean 1,384, median 1,406), corresponding to between 3.1% and 7.3% of the dataset; no individual subject therefore dominated the training pool under LOSO, where 92.7%–96.9% of the data are used for training at each fold. Per-subject class balance was, by contrast, highly heterogeneous: proportions of eating IUs ranged from 7.5% (subject 14) to 66.3% (subject 01), and per-subject imbalance ratios spanned 0.51: 1 to 12.4: 1, reflecting natural variability in eating pace and meal length. Complete per-subject counts are reported in S1 Table.

### 3.2 Machine learning model performance

Table 2 presents the LOSO cross-validation results for all eight machine-learning classifiers, each evaluated under the same LOSO protocol with subject-level cluster-bootstrap 95% confidence intervals (B = 1,000, seed = 1812). Among the machine-learning approaches, XGBoost with hyperparameter tuning achieved the numerically highest balanced accuracy and AUC (Table 2). Because an autonomous smartwatch would not have meal, food, or menu information available at prediction time, we take as the headline model the sensor-only XGBoost, fitted on the 59 motion-derived predictors after removal of the study-arm, meal, and food variables: it achieved balanced accuracy 0.639 [0.607, 0.668] and AUC 0.699 [0.653, 0.743], within the confidence intervals of the full-feature model (Table 2; S1 Appendix Table S1.2 in S1 Appendix), and it remained the numerically best classifier on both metrics. The two feature sets gave near-identical performance, so the contextual variables contributed little and the headline result rests on wrist motion alone; the menu-assignment sub-group analysis (Section 3.6) does not substitute for this ablation, because it does not remove food and meal from the feature matrix. Hyperparameter tuning substantially improved XGBoost’s sensitivity, from 0.417 to 0.593, compared to default parameters, though with a trade-off in specificity. Random Forest showed high specificity (0.931) but poor sensitivity (0.224), indicating a conservative classification bias. LightGBM performed similarly to XGBoost with default parameters but did not benefit from hyperparameter tuning.

To assess whether the differences among classifiers are larger than would be expected from subject-level sampling variability alone, we performed a paired, subject-level cluster bootstrap on the final per-subject metrics and adjusted the resulting p-values for multiplicity across the family of comparisons using the Benjamini and Hochberg false-discovery-rate procedure and the more conservative Holm procedure. Because the subject is the unit of independent replication, the effective sample size for these comparisons is 19, and we base our interpretation on effect sizes and confidence intervals rather than on p-values alone. On balanced accuracy, tuned XGBoost is the numerically best model and is statistically indistinguishable from Logistic Regression (difference 0.009, adjusted p = 0.435), while its advantage over the Decision Tree is borderline (difference 0.027, significant under the false-discovery-rate adjustment but not under Holm). Its advantage over the conservative tree ensembles, the Transformer and the default Attention model, and untuned XGBoost is larger and survives both adjustments, with differences in balanced accuracy ranging from 0.028 to 0.065. The full multiplicity-adjusted comparison across all four metrics is reported in S1 Appendix Table S1.3 in S1 Appendix.

### 3.3 Time series and deep learning results

Attention-based feature extraction with MLP classification achieved a balanced accuracy of 0.609 [0.587, 0.632] under default hyperparameters (Table 3). With hyperparameter tuning (n_heads = 4, τ = 1.0, hidden = 64, α = 10 ⁻ ^3^, lr = 10 ⁻ ⁴), balanced accuracy improved to 0.620 [0.593, 0.644]. The Transformer encoder, with a balanced-accuracy-optimised decision threshold of θ = 0.30, achieved the numerically highest sensitivity among all eleven classifiers (0.691 [0.637, 0.735]) but at the cost of the lowest specificity (0.504 [0.456, 0.549]). Under fully nested threshold selection this sensitivity is 0.649 (S1 Appendix), and after multiplicity adjustment the sensitivity advantage of the Transformer over tuned XGBoost is not statistically significant, while its specificity remains significantly lower (S1 Appendix). Threshold optimization and model ensembling did not improve upon tuned XGBoost.

### 3.4 Inter-subject variability

Substantial variability was observed across subjects. Some subjects (e.g., subjects 04, 12) showed sensitivity above 0.80, while others (e.g., subjects 08, 09, 11) showed sensitivity below 0.30. This inter-subject variability (coefficient of variation > 50% for sensitivity) represents a fundamental challenge for eating detection systems and explains the gap between within-subject and LOSO performance. Per-subject Information Unit counts were essentially uncorrelated with the per-subject sensitivity of the best-performing classifier (tuned XGBoost): Spearman ρ = −0.16, p = 0.51, confirming that the between-subject dispersion of performance metrics reported above is not driven by differences in the amount of data contributed by each participant. S1 Fig provides a forest-style plot of per-subject sensitivity for all eleven classifiers evaluated under LOSO.

### 3.5 Labelling reliability and label-noise robustness

At the IU level (δs = 5 s, 26,304 IUs over the 19 LOSO subjects), the two independent raters agreed on the eating/ non-eating summary label for 82.2% of IUs (73.5% of IUs on non-eating; 8.7% on eating); they disagreed on 4,695 IUs (17.8%), in which one rater but not the other labelled the majority of raw samples as ‘eating’. Computed directly from the two rater streams, Cohen’s kappa at the IU level was 0.41, moderate agreement on the Landis and Koch scale, with substantial between-subject heterogeneity (per-subject agreement 44% to 97%; per-subject kappa 0.09 to 0.86; S1 Appendix Table S1.4 in S1 Appendix). The disagreement was systematic rather than symmetric: of the contested IUs, 94% were labelled eating by the more inclusive rater alone, so the eating-union label tracks that rater and the eating-intersect label the more conservative one. At the raw-frame (5 Hz) level the two raters agreed on 87.3% of the 133,040 frames, with kappa 0.65 (substantial agreement); the higher frame-level value indicates that the moderate IU-level kappa reflects the stricter eating prevalence after majority-vote windowing and the concentration of the residual disagreement at the boundaries of eating episodes, rather than poor labelling. The subjects with the lowest kappa are the same ones that dominate the low-sensitivity tail of the per-subject performance distribution (§3.4 and S1 Fig), indicating that (for those participants) the limitation is not only inter-individual variability of the motion signal but also the ambiguity of the labelling boundary itself.

To bound the influence of label-boundary noise on the reported performance, we refit the best-performing classifier (tuned XGBoost) with the Eating Intersect label (EI = 1 only if both raters assigned the IU to ‘eating’) in place of the Eating Union (EU), following the identical Latin-hypercube tuning and LOSO evaluation protocol of §2.5 (S5 Table, Panel B). Moving from EU to EI (which removes the 4,955 contested EU-only IUs from the positive class) raises the LOSO balanced accuracy from 0.642 [0.612, 0.671] to 0.671 [0.636, 0.702] and the AUC from 0.712 [0.671, 0.749] to 0.753 [0.711, 0.792]. Specificity rises from 0.692 to 0.814, consistent with the contested IUs having indeed been an important source of false positives against a strict label; the sensitivity reduction (0.593 → 0.528) reflects primarily the three-fold shrinkage of the positive class (7,080 → 2,125 eating IUs) rather than a deterioration of model quality, as evidenced by the AUC gain of 0.041. Eating Intersect is a stricter target definition, not merely a cleaner version of Eating Union: the higher performance under Eating Intersect shows that events on which both raters agreed are easier to classify and does not by itself establish that the Eating Union label noise acts only as an attenuating factor. We therefore retain Eating Intersect as a label-robust benchmark rather than as evidence about the direction of the label noise, and the directional conclusions of the study are unchanged by the choice of target.

### 3.6 Robustness to menu assignment

Participants in the NOTION study were randomised to one of two fixed standardised menus (Menu A, 10 subjects; Menu B, 9 subjects after exclusion of subject 02). The two menus comprise different food items across four meal occasions, so if the classifier had specialised on the particular food items of one menu we would expect an arm-dependent performance gap. Stratifying the LOSO metrics of the best-performing model (tuned XGBoost) by menu assignment yielded, under cluster-bootstrap 95% CIs (B = 1000, seed = 1812): Menu A balanced accuracy 0.634 [0.582, 0.680] and AUC 0.710 [0.637, 0.779]; Menu B balanced accuracy 0.651 [0.621, 0.682] and AUC 0.714 [0.671, 0.757]. All pairwise between-menu differences are compatible with zero (balanced accuracy Δ = −0.017 [−0.081, + 0.039], p = 0.560; AUC Δ = −0.004 [−0.082, + 0.080], p = 0.992; sensitivity Δ = −0.031, p = 0.714; specificity Δ = −0.003, p = 0.970), indicating that the classifier generalises across the two sets of food items within our Italian/Western eating style.

### 3.7 Few-shot personalisation of the best-performing classifier

Given the substantial inter-subject variability identified in §3.4 and the inter-rater reliability differences highlighted in §3.5, we explored whether a light-weight personalisation step (fine-tuning the tuned XGBoost classifier on a small set of subject-specific labelled samples) could recover performance on the low-sensitivity tail of the per-subject distribution. We considered the five difficult subjects (the bottom five by baseline LOSO sensitivity under XGBoost with default hyperparameters: subjects 08, 09, 10, 11, 16; baseline sensitivity range 0.16 to 0.29, mean 0.21) and the remaining fourteen subjects.

An earlier version of this experiment drew calibration Information Units at random from each subject’s held-out fold, which, given the one-second stride, allowed the calibration and the evaluation windows to share raw signal, and its feature set had retained the closely related Eating Intersect label; the meal-blocked, sensor-only protocol described in §2.5.8 removes both. Under this protocol the benefit was modest and variable: on the five difficult subjects the mean improvement in sensitivity ranged from about 0.014 to 0.059 across the numbers of calibration examples and was not monotonic, and at 100 examples it was about 0.050 (mean sensitivity rising from 0.278 to 0.328); on the remaining 14 subjects it was at most about 0.037 and was 0.023 at 100 examples. The effect on the difficult subjects was driven mainly by a single participant (subject 09, from 0.172 to 0.313), and the per-subject test sets were small, so the individual estimates are uncertain. We therefore present personalisation as a modest, exploratory direction rather than as evidence of deployable personalisation, and we do not treat it as the single most important avenue for improvement. The per-group and per-subject results are reported in S1 Appendix Table S1.5 in S1 Appendix.

## 4. Discussion

Interest in wearable sensors for tracking human activities has grown substantially [33,34]. Automatic detection of food intake is necessary for gaining insights into eating behavior and addressing the limitations of self-reported dietary data [23]. Many studies have focused on eating detection, exploring novel sensors placed on the neck, head, ear, and wrist, utilizing various detection modalities such as acoustic [35–38], inertial [13,14,24,39], visual [40,41]. However, despite significant progress, many of the techniques developed so far rely on multiple on-body sensors, making them impractical for everyday use [24].

The results of this study reveal important insights into the challenges of eating detection with wrist-worn sensors, as evaluated through rigorous subject-independent validation. Although tuned XGBoost achieves the numerically highest balanced accuracy and AUC under leave-one-subject-out cross-validation (Table 2), the multiplicity-adjusted paired comparison (S1 Appendix) makes clear that no single architecture is best on every metric. On balanced accuracy, Logistic Regression (a linear baseline) is statistically indistinguishable from tuned XGBoost (difference 0.009, adjusted p = 0.435), and the Decision Tree is borderline; the other models in that comparison have lower balanced accuracy by margins that survive multiplicity adjustment. On sensitivity, the Transformer is numerically higher than tuned XGBoost, but under nested threshold selection and multiplicity adjustment this difference is not statistically significant (difference 0.055, with a confidence interval that runs from slightly below zero to about 0.12, adjusted p = 0.18), whereas its specificity is significantly lower, by about 0.16 (confidence interval 0.10 to 0.22, p < 0.001). Practically, this means that the choice of classifier should be driven by the clinical or application-level trade-off between missed eating episodes and false positives: tuned XGBoost offers the best aggregate trade-off, the Transformer shifts the operating point towards higher sensitivity at a significant cost in specificity but without a statistically robust gain, and Logistic Regression provides a simple, interpretable, almost-as-accurate alternative for settings in which transparency of the decision process matters. These results are substantially lower than the metrics (>0.80) reported in studies using random train-test splits [8,25], including those finding Random Forest superior to XGBoost [42,43]. The discrepancy reflects a methodological artifact: when subject identity leaks into training data through random splitting, models exploit individual signatures (handedness, eating speed, utensil preferences) rather than learning generalizable eating patterns [11,44]. Our per-subject analysis confirms this: sensitivity ranged from above 0.80 to below 0.30 across individuals, identifying inter-subject variability as the primary limiting factor for real-world deployment.

The moderate performance of our models (balanced accuracy around 0.64) should be interpreted in the context of task difficulty and experimental design. Studies reporting higher performance typically address different tasks: Kyritsis et al. [32] achieved F1 = 0.923 for bite detection within already-identified meal sessions using end-to-end deep learning on the Food Intake Cycle (FIC) dataset, while Stankoski et al. [8] reported F1 = 0.82 for eating segment detection using a sophisticated pipeline combining deep learning with HMM post-processing and careful data selection strategies. In contrast, our task (classifying eating versus non-eating across all recorded time using traditional ML on hand-crafted features) is more challenging. Thomaz et al. [24], addressing a task more comparable to ours, reported F-scores ranging from 0.60 to 0.76 depending on temporal evaluation granularity when testing on free-living data. Additionally, our 5 Hz sampling rate (commercial smartwatch limitation) may miss rapid movements captured by research-grade sensors operating at 25–100 Hz. These factors suggest that balanced accuracy around 0.60-0.65 may be realistic for window-based eating classification from low-frequency commercial sensors in semi-naturalistic conditions.

A key contribution of this study is the use of a commercially available smartwatch, the Garmin Fenix 5, for dietary monitoring. Unlike previous studies relying on specialized or custom-built sensors, using an off-the-shelf device enhances feasibility for large-scale deployment and real-world adoption [4,6]. Commercial smartwatches offer a practical and cost-effective solution for continuous dietary monitoring, with built-in motion sensors to capture wrist motion during eating episodes [45]. Several studies have highlighted the potential of commercial wearable devices in health monitoring applications, ranging from physical activity tracking to sleep pattern analysis [9,10]. The widespread availability of such devices makes them an attractive option for scalable dietary assessments in diverse populations [11].

Our evaluation of deep learning approaches yielded mixed results. The Transformer encoder, with its balanced-accuracy-optimised decision threshold (θ = 0.30; §2.5.4), achieved the numerically highest sensitivity among the eleven classifiers (0.691 [0.637, 0.735]); however, under nested threshold selection and multiplicity-adjusted comparison this advantage over tuned XGBoost is not statistically significant (S1 Appendix), and it came at the cost of the lowest specificity (0.504 [0.456, 0.549]), indicating a tendency toward false positives. This sensitivity-specificity trade-off is common in imbalanced classification problems [46] and may be acceptable in applications where missing eating events is more costly than false alarms. Attention pooling with an MLP classifier provided a middle ground: at default hyperparameters, it recorded sensitivity 0.521 [0.471, 0.574] and specificity 0.698 [0.660, 0.736]; after tuning, sensitivity 0.577 [0.509, 0.652] and specificity 0.662 [0.611, 0.712] (Table 3).

We tested our method on data collected under semi-naturalistic conditions rather than in controlled laboratory settings with scripted eating protocols. Most previous studies have conducted experiments in artificial environments, where participants follow pre-defined eating protocols [12,13,37,47]. However, semi-naturalistic scenarios present additional challenges, such as spontaneous eating behaviors, variations in food choices, and environmental distractions [14]. Our study contributes to bridging this gap by testing the proposed classification models in realistic settings, demonstrating their robustness and applicability in daily life. The importance of free-living dietary monitoring has been emphasized in prior research, as it better reflects habitual eating behaviors and provides more ecologically valid data [16,17]. By capturing data in such settings, this study provides valuable insights into the complexities of dietary behaviors and the potential of wearable technology for long-term monitoring [18].

The balanced accuracy of our best-performing classifier, 0.639 [95% CI 0.607, 0.668] for the sensor-only model and 0.642 [0.612, 0.671] for the full-feature model under the Eating Union target, and 0.671 [0.636, 0.702] under the label-robust Eating Intersect target, is not yet sufficient for clinical-grade deployment such as real-time monitoring of patients with eating disorders, where sensitivity and specificity above 0.85 are typically required [6]. It may, however, be more relevant to lower-stakes settings than to clinical monitoring: for example, research uses in which the classifier serves as an aggregate-level predictor rather than an individual decision tool, or wellness applications in which the detector provides loose, uncertainty-aware feedback to the end user. Establishing usable performance in either setting would still require external validation in broader populations and on additional devices. Our own analyses identify a set of complementary strategies that may help close the remaining performance gap. First, few-shot personalisation (§3.7), under a leakage-free, meal-blocked protocol, yields a modest and variable improvement in sensitivity on the low-performing tail of subjects, of the order of 0.05 with about 100 user-specific labelled Information Units; we regard this as exploratory rather than established. Second, enlarging the training corpus is required: the retrospective minimum-detectable-effect analysis (§4 Limitations) shows that reliably distinguishing classifiers at a ~ 5-percentage-point resolution requires roughly N = 100 subjects. Third, the 5 Hz sampling rate of the Garmin Fenix 5 is a known lower bound on achievable discrimination [24,32]: research-grade IMUs at 25–100 Hz resolve intra-gesture structure that is invisible at 5 Hz. Fourth, multimodal fusion is the natural complement to pure-motion classification in the regimes where wrist motion alone is ambiguous, as quantified by the false-positive-pattern analysis (approximately 70% of false positives isolated from the nearest eating event). Three families of modalities have documented value: *acoustic chewing sensing* via piezoelectric neck sensors [35], earpad microphones [36,48], or paired Bluetooth-headset audio [38]; *proximity and contextual fusion* [45] which can be harvested with a phone-companion architecture without changing the wrist-side instrumentation; and *other physiological signals* such as heart rate, which remain speculative for this application. Fifth, the label-robust Eating Intersect target (§3.5) yields a higher balanced accuracy (higher by 0.029) and AUC (higher by 0.041) than the Eating Union, reflecting that events agreed on by both raters are easier to classify.

The classification results reported in this paper are obtained by an offline evaluation of the LOSO cross-validation folds. The computational envelope of the best-performing classifier, however, is well within the budget of a real-time operation: a benchmark of the tuned XGBoost model (companion repository, *inference_latency.csv*) records a median single-Information-Unit inference latency of 4.6 ms on a commodity single-threaded CPU (95th percentile 7.3 ms; per-IU latency in a 1000-IU batch 12 µs; throughput ~85,000 IU/s; serialised model size 3.7 MB). Three deployment architectures are standard in the wrist-worn eating-detection literature: *on-watch* inference, which would require model compression to fit within the ~ 128 kB application-available memory of the Garmin Connect IQ SDK; *phone-companion* inference, in which raw sensor data is forwarded over Bluetooth LE to a paired smartphone for inference (the architecture adopted by Kyritsis et al. [25], with end-to-end latency typically dominated by the BLE transport at 100–300 ms); and *cloud-offloaded* inference for non-strict real-time dietary-phenotyping applications. Sustained monitoring amplifies the energy-budget concerns. The Garmin Fenix 5 advertises up to two weeks of battery life in standard smartwatch mode without continuous third-party sensing; enabling the RawLogger API at 5 Hz keeps the IMU continuously active and writes on the order of 1 MB/hour of raw data, shortening effective battery life to a few days on a full charge, consistent with a diurnal deployment with overnight recharging, but not with continuous multi-week sensing. Three energy-efficiency strategies are established: (i) *duty-cycled sensing*, activating the high-rate IMU only during candidate eating windows detected by a lightweight context signal [45,49]; (ii) *cascaded inference*, keeping a compact gatekeeper continuously active and invoking the full classifier only when the gatekeeper fires; and (iii) *edge buffering with periodic burst-sync*. We did not measure battery drain during the NOTION protocol; on-device measurement over multi-day deployments is a natural follow-up.

### 4.1 Limitations

Several limitations should be acknowledged. First, the sample size (n = 20 subjects) limits statistical power and generalizability. With only 19 subjects for LOSO validation, each fold’s test set represents a single individual, leading to high variance in per-subject metrics. Second, data collection occurred under semi-naturalistic conditions but still involved predefined meals, which may not fully capture the variability of free-living eating behaviors. Third, the class imbalance (26.9% eating) required careful handling through class weights, but optimal strategies for imbalanced eating data remain an open question [8]. Fourth, the 5 Hz sampling rate of commercial smartwatches may miss rapid movements captured by research-grade IMUs operating at 50–100 Hz [24]. Additionally, variability in eating styles and utensils introduces heterogeneity in the motion data. Cultural differences in eating behaviors can significantly impact gesture recognition accuracy, requiring more adaptable and culturally sensitive models [14]. In addition, the two evaluators differed systematically in how readily they labelled eating: one rater labelled eating roughly two and a half times as often as the other, so the eating-union and eating-intersect targets bracket this rater disagreement rather than removing it (Methods §2.5.7, §3.5, S1 Appendix Table S1.4 in S1 Appendix).

In addition to the points above, the NOTION dataset itself represents only a narrow slice of the real-world variability of eating behaviour. Participants were 19 healthy Italian adults aged 20–30 years consuming standardised Western-style meals in a university cafeteria with conventional utensils (fork, knife, spoon); the dataset does not include children or older adults, cultural eating styles that rely on chopsticks, hand-feeding, or bowl-held consumption, or eating under pathological conditions. The internal robustness check reported in §3.6 indicates that, at least within the Italian/Western cuisine pattern of our dataset, the classifier does not depend on the particular set of food items consumed; cross-cultural generalisation, however, remains open.

The Garmin Fenix 5 RawLogger API exposes the inertial measurement unit at 5 Hz, which sets the temporal ceiling of the present analysis. The dominant temporal scale of an eating gesture (approximately 3–5 seconds for a complete cycle of hand-to-mouth, chewing and return [14,24]) corresponds to a fundamental frequency of ~0.2–0.3 Hz, well below the 2.5 Hz Nyquist limit of our acquisition and therefore captured without aliasing. By contrast, fine-grained intra-gesture structure such as rapid pronation, micro-tremor and chewing-rate modulation contains frequency components in the 3–10 Hz range and is under-sampled at 5 Hz. Higher-rate wrist-worn IMUs in comparable studies acquire at 25 Hz [24], 60 Hz [14], or ≈62 Hz [32], and their balanced accuracies or F-scores on cognate tasks (typically 0.75–0.90) are systematically higher than the 0.64 we report on 5 Hz commercial hardware; the sampling-rate ceiling is therefore plausibly part of the performance gap. We nevertheless selected the Fenix 5 deliberately as a widely-deployed consumer smartwatch: the performance we report should be interpreted as a *commercially-achievable* baseline, complementary to the higher-rate research-grade work cited above.

The NOTION dataset was collected while participants consumed standardised meals in a semi-naturalistic cafeteria, without structured conversation. The training set therefore covers non-eating wrist motions that occur spontaneously during a seated meal (cutlery manipulation, food handling, wiping the mouth, resting between bites, hand-on-face, small postural adjustments) but does not include the broader spectrum of hand-to-mouth behaviours characteristic of non-meal daily life, such as gesturing during a conversation, grooming, or using a smartphone while walking. A per-Information-Unit analysis of the best-performing classifier (tuned XGBoost, companion repository, *fp_pattern_per_subject.csv*) indicates that about 70% of its false-positive IUs occur in temporal isolation from any eating event (> 10 s from the nearest true eating IU); the remaining ~30% are boundary-adjacent (within ±10 s of an eating event), and this boundary-adjacent fraction is statistically over-represented relative to the true-negative pool (17.5% of false positives vs 9.0% of true negatives at the tighter ±5 s window, enrichment ratio 1.95; 29.8% vs 19.4% at ±10 s, enrichment ratio 1.54), consistent with labelling-boundary noise (§3.5). Generalisation to 24-hour free-living data remains to be validated in a dedicated study.

Two sources of sensor-placement variability deserve explicit comment. First, hand dominance is controlled by construction rather than evaluated empirically: our feature set is extracted from the right wrist (§2.3) and the 19 retained participants are all right-handed, so the right wrist coincides with the dominant and eating hand for every retained subject. The single left-handed participant (subject 02) was excluded (§2.1); with a single left-handed subject, no formal contrast between dominant- and non-dominant-wrist placement was possible. Second, watch-strap tightness was not recorded during the NOTION protocol: residual between-subject variability in strap tightness may contribute to the inter-subject spread of the per-subject metrics reported in §3.4 and S1 Fig.

The deliberate feature-level denoising choice (§2.3) is validated by the ablation reported in S6 Table: applying a 2nd-order zero-phase Butterworth 0.3 Hz low-pass filter upstream of the window aggregation produces a statistically significant loss of sensitivity (Δ = −0.033, p = 0.002) without compensating gains in balanced accuracy or AUC, confirming that the implicit denoising provided by the 5-second window statistics preserves more eating-relevant information than a hard low-pass stage on our 5 Hz data. An exploration of alternative cut-off frequencies on purpose-collected higher-sampling-rate data remains a natural follow-up.

The sensitivity confidence interval of the best-performing classifier has a half-width of approximately 0.08 (roughly 13% of the point estimate), reflecting the precision achievable with 19 subject-level replications. A retrospective power analysis using the between-subject SDs of our best-performing classifier indicates that, at N = 19, we are powered (α = 0.05, 1 − β = 0.80) to detect sensitivity differences of ~0.12 and balanced-accuracy differences of ~0.05; discriminating finer differences would require datasets of order 100 subjects.

The findings of this study contribute to the growing field of automated dietary assessment and have significant implications for public health monitoring and nutritional interventions. Wearable-based eating detection systems offer a scalable and objective alternative to traditional self-reported dietary assessment methods, which are prone to inaccuracies and biases.

Future research should focus on several directions. The most immediate priority is external validation on datasets with broader demographic and cultural coverage. Three public benchmarks are natural candidates: the FIC dataset [32], which covers Greek Mediterranean eating at ≈62 Hz on a Microsoft Band 2; the Clemson Cafeteria dataset [14,50], which includes 271 participants across structured meal settings; and the OREBA dataset [51], which spans individual and communal eating (100 and 102 participants in the discrete- and shared-dish scenarios). Cross-dataset comparison, however, is not a trivial drop-in: the research-grade IMUs used in those studies sample at 25–100 Hz, their labelling granularities differ, and their sensor placements introduce additional heterogeneity. Harmonising these protocols is therefore the necessary scaffolding for a meaningful external-validation study. Implementation and on-device evaluation of a real-time pipeline (with specific attention to streaming feature extraction, BLE buffering and end-to-end latency measurement on the target hardware) is a natural next step; prior implementations in the same application domain [3,25,45] suggest that end-to-end latencies in the 100–300 ms range are achievable. Empirical validation of multimodal fusion will require a data-collection campaign that combines wrist motion with a paired modality, because the NOTION dataset was not instrumented for audio or paired-phone streams. An exploration of alternative filter cut-off frequencies on purpose-collected higher-sampling-rate data, where the DSP trade-off is genuinely informative, is a natural extension of the S6 Table ablation. Semi-supervised and self-supervised learning approaches may help leverage the abundance of unlabeled wearable data [52]. Building on the few-shot personalization experiment reported in §3.7, which under a leakage-free, meal-blocked protocol produced a modest improvement of about 0.05 in sensitivity on the low-performing tail of subjects with about 100 user-specific labelled Information Units, a personalization pipeline (on-device fine-tuning, user-in-the-loop label elicitation, drift-aware re-calibration) is one possible translational direction [53].

## 5. Conclusions

This study evaluated eating detection from commercial smartwatch sensors using rigorous Leave-One-Subject-Out cross-validation. Our best model, XGBoost with hyperparameter tuning using motion signals only, achieved balanced accuracy of 0.639 [95% CI 0.607, 0.668], sensitivity of 0.594 [0.528, 0.663], and specificity of 0.683 [0.630, 0.732]; the full-feature model that additionally used experimental-context variables performed near-identically (Table 2 and S1 Appendix).These results are substantially lower than those reported in studies using less stringent validation strategies, highlighting the importance of subject-independent evaluation for realistic performance assessment.

Paired subject-level comparisons, adjusted for multiplicity, indicate that Logistic Regression performed comparably to tuned XGBoost on balanced accuracy (S1 Appendix), while the Transformer encoder achieved a numerically higher sensitivity that was not statistically significant after nested threshold selection and multiplicity adjustment, at the cost of significantly reduced specificity. The high inter-subject variability observed (CV > 50% for sensitivity) represents a fundamental challenge for wrist-based eating detection and suggests that personalization or larger training datasets will be necessary for practical deployment. Our findings underscore that reported performance metrics in eating detection research depend critically on validation methodology, and future studies should prioritize subject-independent evaluation to enable meaningful comparison across methods.

## Supporting information

S1 Fig Per-subject sensitivity under Leave-One-Subject-Out (LOSO) cross-validation. Forest-style display of the sensitivity achieved on each held-out subject by the eleven classifiers evaluated: Logistic Regression, Decision Tree, Random Forest (default and hyperparameter-tuned), XGBoost (default and hyperparameter-tuned), LightGBM (default and hyperparameter-tuned), Attention pooling (default and hyperparameter-tuned; decision threshold 0.50), and Transformer encoder (decision threshold 0.30, selected by balanced-accuracy grid search on LOSO predictions; see Methods §2.5.4). Each point is the sensitivity observed when the corresponding subject served as the test fold; the subject identifier is printed next to the point. Subjects are ranked within each panel by ascending sensitivity (y axis = rank from 1 to 19), so that the visual spread across the y axis reflects the inter-individual variability of each classifier. The vertical dashed line marks the 0.5 chance reference; the vertical dotted red line denotes the mean-across-subjects sensitivity for that classifier.
(TIFF)

S1 Table Per-subject Information Unit counts under LOSO cross-validation (δs = 5 s). For each of the 19 LOSO subjects, the table reports the total number of Information Units (IUs) contributed; the breakdown into eating and non-eating IUs; the per-subject proportion of eating IUs; the non-eating-to-eating imbalance ratio; the share of the full 26,304-IU dataset; and the LOSO training size when that subject was held out. Subject 02 (left-handed) is excluded.
(DOCX)

S2 Table Architectures and parameter counts of the time-series classifiers. Side-by-side specification of the Attention pooling + MLP and Transformer encoder models. Complements Methods §2.5.4.
(DOCX)

S3 Table Window-size sensitivity analysis under LOSO (XGBoost, default hyperparameters). Cluster-bootstrap 95% CIs (B = 1000, seed = 1812) over 19 LOSO subjects.
(DOCX)

S4 Table Complete list of 75 predictors fed to the ML classifiers (δs = 5 s). (DOCX)

S5 Table Inter-rater reliability and label-noise robustness. Panel A: IU-level inter-rater agreement (δs = 5 s; 19 subjects; 26,304 IUs), computed directly from the two individual rater streams. Panel B: XGBoost (tuned) LOSO performance under the Eating Union (EU) and Eating Intersect (EI) targets.
(DOCX)

S6 Table Filter-vs-no-filter ablation (Butterworth 0.3 Hz vs window-aggregation denoising). Same raw 5 Hz stream, 19 subjects, 26,058 IUs, 27.0% eating, same XGBoost-tuned hyperparameters. Panel A: marginal LOSO performance of each pipeline. Panel B: paired cluster-bootstrap comparison (filtered minus unfiltered).
(DOCX)

S1 Appendix Supplementary analyses. Fully nested subject-level cross-validation and the post-selection optimism gap (Table S1.1); full versus sensor-only feature-set ablation (Table S1.2); multiplicity-adjusted pairwise model comparisons across the four metrics (Table S1.3); inter-rater agreement at the Information-Unit and raw-frame levels, with the exact rater-by-rater contingency table and Cohen’s kappa (Table S1.4); and the meal-blocked few-shot personalisation experiment (Table S1.5). Also includes the companion-repository data-file inventory and references.
(DOCX)
