# Supplementary figures and images for: Recognition of eating episodes via commercial smartwatch sensors analysis
Source: PLOS Digit Health. 2026 Jul 7;5(7):e0001539. doi: 10.1371/journal.pdig.0001539 (PMC13340811; doi:10.1371/journal.pdig.0001539)

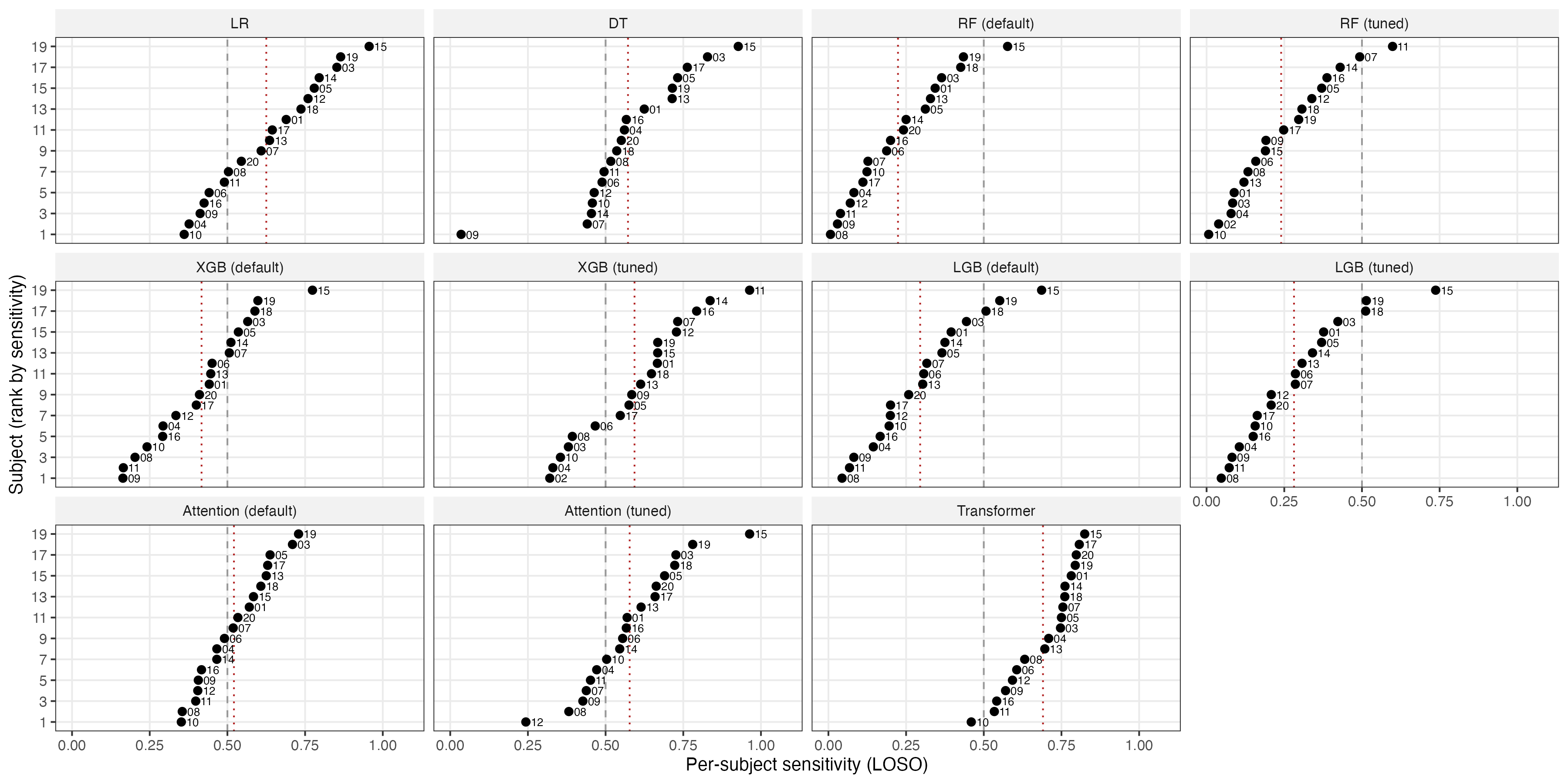

Supplement: S1 Fig — Forest-style display of the sensitivity achieved on each held-out subject by the eleven classifiers evaluated: Logistic Regression, Decision Tree, Random Forest (default and hyperparameter-tuned), XGBoost (default and hyperparameter-tuned), LightGBM (default and hyperparameter-tuned), Attention pooling (default and hyperparameter-tuned; decision threshold 0.50), and Transformer encoder (decision threshold 0.30, selected by balanced-accuracy grid search on LOSO predictions; see Methods §2.5.4). Each point is the sensitivity observed when the corresponding subject served as the test fold; the subject identifier is printed next to the point. Subjects are ranked within each panel by ascending sensitivity (y axis = rank from 1 to 19), so that the visual spread across the y axis reflects the inter-individual variability of each classifier. The vertical dashed line marks the 0.5 chance reference; the vertical dotted red line denotes the mean-across-subjects sensitivity for that classifier. (TIFF) [file pdig.0001539.s001.tiff]
