# Supplementary material for: Recognition of eating episodes via commercial smartwatch sensors analysis
Source: PLOS Digit Health. 2026 Jul 7;5(7):e0001539. doi: 10.1371/journal.pdig.0001539 (PMC13340811; doi:10.1371/journal.pdig.0001539)
Supplement: S1 Table — For each of the 19 LOSO subjects, the table reports the total number of Information Units (IUs) contributed; the breakdown into eating and non-eating IUs; the per-subject proportion of eating IUs; the non-eating-to-eating imbalance ratio; the share of the full 26,304-IU dataset; and the LOSO training size when that subject was held out. Subject 02 (left-handed) is excluded. (DOCX) [file pdig.0001539.s002.docx]

## S1 Table. Per-subject Information Unit counts under LOSO cross-validation (δs = 5 s).

For each of the 19 LOSO subjects, the table reports the total number of Information Units (IUs) contributed; the breakdown into eating and non-eating IUs; the per-subject proportion of eating IUs; the non-eating-to-eating imbalance ratio; the share of the full 26,304-IU dataset; and the LOSO training size when that subject was held out. Subject 02 (left-handed) is excluded.

| Subject | Total IU | Eating IU | Non-eating IU | Prop. eating | Imbalance ratio (NE:E) | Share of dataset | LOSO training size |
| --- | --- | --- | --- | --- | --- | --- | --- |
| 01 | 1,229 | 815 | 414 | 66.3% | 0.51 | 4.67% | 25,075 |
| 03 | 1,626 | 826 | 800 | 50.8% | 0.97 | 6.18% | 24,678 |
| 04 | 1,459 | 560 | 899 | 38.4% | 1.61 | 5.55% | 24,845 |
| 05 | 1,611 | 1,048 | 563 | 65.1% | 0.54 | 6.12% | 24,693 |
| 06 | 1,453 | 528 | 925 | 36.3% | 1.75 | 5.52% | 24,851 |
| 07 | 1,406 | 322 | 1,084 | 22.9% | 3.37 | 5.35% | 24,898 |
| 08 | 1,005 | 296 | 709 | 29.5% | 2.40 | 3.82% | 25,299 |
| 09 | 1,496 | 342 | 1,154 | 22.9% | 3.37 | 5.69% | 24,808 |
| 10 | 1,398 | 435 | 963 | 31.1% | 2.21 | 5.31% | 24,906 |
| 11 | 973 | 206 | 767 | 21.2% | 3.72 | 3.70% | 25,331 |
| 12 | 1,564 | 341 | 1,223 | 21.8% | 3.59 | 5.95% | 24,740 |
| 13 | 1,930 | 280 | 1,650 | 14.5% | 5.89 | 7.34% | 24,374 |
| 14 | 1,181 | 88 | 1,093 | 7.5% | 12.42 | 4.49% | 25,123 |
| 15 | 814 | 137 | 677 | 16.8% | 4.94 | 3.09% | 25,490 |
| 16 | 984 | 120 | 864 | 12.2% | 7.20 | 3.74% | 25,320 |
| 17 | 1,381 | 135 | 1,246 | 9.8% | 9.23 | 5.25% | 24,923 |
| 18 | 1,325 | 209 | 1,116 | 15.8% | 5.34 | 5.04% | 24,979 |
| 19 | 1,774 | 214 | 1,560 | 12.1% | 7.29 | 6.74% | 24,530 |
| 20 | 1,695 | 178 | 1,517 | 10.5% | 8.52 | 6.44% | 24,609 |
| **Total** | **26,304** | **7,080** | **19,224** | **26.9%** | **2.72** | 100% |  |
| Mean (SD) | 1,384 (296) | 373 (270) | 1,012 (335) | 26.6% | 4.47 |  |  |
| Median [min, max] | 1,406 [814, 1,930] | 296 [88, 1,048] | 963 [414, 1,650] | 21.8% [7.5%, 66.3%] | 3.59 [0.51, 12.42] |  |  |
