# Supplementary material for: Recognition of eating episodes via commercial smartwatch sensors analysis
Source: PLOS Digit Health. 2026 Jul 7;5(7):e0001539. doi: 10.1371/journal.pdig.0001539 (PMC13340811; doi:10.1371/journal.pdig.0001539)
Supplement: S2 Table — Side-by-side specification of the Attention pooling + MLP and Transformer encoder models. Complements Methods §2.5.4. (DOCX) [file pdig.0001539.s003.docx]

## S2 Table. Architectures and parameter counts of the time-series classifiers.

Side-by-side specification of the Attention pooling + MLP and Transformer encoder models. Complements Methods §2.5.4.

| Aspect | Attention pooling + MLP | Transformer encoder |
| --- | --- | --- |
| **Input** | 25 timesteps × 6 channels (acc_x, acc_y, acc_z, pitch, roll, power) | Same |
| **Feature representation** | Deterministic multi-head attention pooling: 4 heads with *hand-crafted* energy-based, axis-based, gradient-based, and uniform weighting; softmax τ = 1.0. No learnable attention parameters. | End-to-end learnable attention (nn.TransformerEncoder) |
| **Dimensionality** | 50 deterministic features | d_model = 64 (learnable projection from 6 channels) |
| **Positional encoding** | n/a | Learnable embedding, 25 × 64 = 1,600 params |
| **Encoder depth** | 1 hidden MLP layer (64 units) | 4 Transformer encoder layers |
| **Attention heads** | 4 (hand-crafted; no parameters) | 4 (learnable multi-head self-attention) |
| **Feed-forward width** | n/a | dim_feedforward = 128 |
| **Aggregation** | Attention-weighted pooling → MLP | Global avg pooling → head (64 → 32 → 1) |
| **Dropout** | L2 α = 10⁻³ | 0.4 (positional, attention, FF, classifier) |
| **Weight decay** | α as weight decay | 10⁻⁴ (AdamW) |
| **Optimizer** | sklearn MLP (Adam, batch = 200, early stop on 10% split) | AdamW + ReduceLROnPlateau (0.5, patience 5) |
| **Learning rate** | 10⁻⁴ | 5 × 10⁻⁴ |
| **Gradient clipping** | n/a | max-norm = 1.0 |
| **Class-imbalance** | Oversampling minority class | BCEWithLogitsLoss pos_weight + WeightedRandomSampler |
| **Early stopping** | On 10% validation split | Training-loss plateau, patience 15 |
| **Trainable parameters** | **3,394** | **138,049** |
| **Params : training samples** | **≈ 0.14 : 1** | **≈ 5.5 : 1** |
