# Supplementary material for: Recognition of eating episodes via commercial smartwatch sensors analysis
Source: PLOS Digit Health. 2026 Jul 7;5(7):e0001539. doi: 10.1371/journal.pdig.0001539 (PMC13340811; doi:10.1371/journal.pdig.0001539)
Supplement: S3 Table — Cluster-bootstrap 95% CIs (B = 1000, seed = 1812) over 19 LOSO subjects. (DOCX) [file pdig.0001539.s004.docx]

## S3 Table. Window-size sensitivity analysis under LOSO (XGBoost, default hyperparameters).

Cluster-bootstrap 95% CIs (B = 1000, seed = 1812) over 19 LOSO subjects.

| δs (s) | N IUs | Sensitivity | Specificity | Balanced accuracy | AUC |
| --- | --- | --- | --- | --- | --- |
| 1 | 26,608 | 0.492 [0.412, 0.574] | 0.782 [0.736, 0.823] | 0.637 [0.604, 0.667] | 0.693 [0.649, 0.735] |
| 2 | 26,532 | 0.488 [0.408, 0.558] | 0.788 [0.744, 0.828] | 0.638 [0.606, 0.668] | 0.706 [0.664, 0.747] |
| 3 | 26,456 | 0.504 [0.418, 0.586] | 0.791 [0.747, 0.833] | 0.648 [0.612, 0.680] | 0.718 [0.676, 0.758] |
| 4 | 26,380 | 0.460 [0.385, 0.537] | 0.804 [0.757, 0.845] | 0.632 [0.604, 0.659] | 0.710 [0.676, 0.745] |
| **5** | **26,304** | **0.417 [0.341, 0.491]** | **0.814 [0.765, 0.858]** | **0.615 [0.589, 0.638]** | **0.693 [0.656, 0.726]** |

*Bold: window size adopted. The δs = 5 s row reproduces the XGBoost (default) entry of Table 2 so that the downstream analysis row is bit-identical to the main-text reference; the remaining four rows are obtained from the dedicated window-size bootstrap (timeframe_ci.csv), which uses the same subject-level resampling protocol and seed (1,812) but aggregates per-fold predictions from the five candidate-δs re-fits. All CIs overlap across δs; max balanced-accuracy difference = 0.033 (δs = 3 vs 5). See Methods §2.4 for justification.*
