# Supplementary material for: Recognition of eating episodes via commercial smartwatch sensors analysis
Source: PLOS Digit Health. 2026 Jul 7;5(7):e0001539. doi: 10.1371/journal.pdig.0001539 (PMC13340811; doi:10.1371/journal.pdig.0001539)
Supplement: S4 Table — (DOCX) [file pdig.0001539.s005.docx]

## S4 Table. Complete list of 75 predictors fed to the ML classifiers (δs = 5 s).

| Category | Count | Features |
| --- | --- | --- |
| Slope (regression coefficient vs time) | 7 | acc_x, acc_y, acc_z, pitch, roll, power, total_energy |
| Mean (right wrist) | 7 | mean_{acc_x,...,total_energy}_right |
| Median (right wrist) | 7 | med_{acc_x,...,total_energy}_right |
| Minimum (right wrist) | 7 | min_{acc_x,...,total_energy}_right |
| Maximum (right wrist) | 7 | max_{acc_x,...,total_energy}_right |
| Inter-quartile range (right wrist) | 7 | iqr_{acc_x,...,total_energy}_right |
| Standard deviation (right wrist) | 7 | sd_{acc_x,...,total_energy}_right |
| Coefficient of variation (right wrist) | 7 | cv_{acc_x,...,total_energy}_right |
| Axis cross-correlation | 3 | cor_xy, cor_yz, cor_zx |
| Arm (dummy) | 1 | arm_b |
| Meal (dummy, 4 levels) | 3 | meal_X02, meal_X03, meal_X04 |
| Food item (dummy, 13 levels) | 12 | food_X02 ... food_X13 |
| **Total** | **75** |  |

*Also provided as feature_list.csv in the companion repository. Time-series classifiers (Table 3) use raw 25 × 6 signals, not these features (§2.5.4, S2 Table).*
