# Supplementary material for: Recognition of eating episodes via commercial smartwatch sensors analysis
Source: PLOS Digit Health. 2026 Jul 7;5(7):e0001539. doi: 10.1371/journal.pdig.0001539 (PMC13340811; doi:10.1371/journal.pdig.0001539)
Supplement: S5 Table — Panel A: IU-level inter-rater agreement (δs = 5 s; 19 subjects; 26,304 IUs), computed directly from the two individual rater streams. Panel B: XGBoost (tuned) LOSO performance under the Eating Union (EU) and Eating Intersect (EI) targets. (DOCX) [file pdig.0001539.s006.docx]

## S5 Table. Inter-rater reliability and label-noise robustness.

### Panel A. IU-level inter-rater agreement (δs = 5 s; 19 subjects; 26,304 IUs), computed directly from the two individual rater streams.

Counts of Information Units (Rater 1 is the more inclusive evaluator):

|  | Rater 2 eating | Rater 2 non-eating | Total |
| --- | --- | --- | --- |
| Rater 1 eating | 2,286 | 4,411 | 6,697 |
| Rater 1 non-eating | 284 | 19,323 | 19,607 |
| Total | 2,570 | 23,734 | 26,304 |

| Statistic | Value |
| --- | --- |
| Percent agreement | 82.2% |
| Cohen's kappa | 0.41 (moderate) |

*Rater 1 labelled eating in 25.5% of Information Units against 9.8% for Rater 2; of the 4,695 contested Information Units, 4,411 (94%) were labelled by Rater 1 alone, so the eating-union label tracks Rater 1 and the eating-intersect label tracks Rater 2. Per-subject: agreement 44% to 97%; kappa 0.09 to 0.86. At the raw-frame (5 Hz) level the agreement is 87.3% and kappa 0.65 (substantial); the full two-level analysis is in S1 Appendix Table S1.4. Full per-subject values in per_subject_agreement.csv.*

### Panel B. XGBoost (tuned) LOSO under EU vs EI targets.

Cluster-bootstrap 95% CIs (B = 1000, seed = 1812). EI re-tuned (trees = 388, depth = 5, lr = 0.019, min_n = 5).

| Target | Sensitivity | Specificity | Balanced accuracy | AUC |
| --- | --- | --- | --- | --- |
| **EU (primary)** | 0.593 [0.512, 0.677] | 0.692 [0.624, 0.756] | 0.642 [0.612, 0.671] | 0.712 [0.671, 0.749] |
| **EI (robust)** | 0.528 [0.446, 0.608] | 0.814 [0.771, 0.856] | 0.671 [0.636, 0.702] | 0.753 [0.711, 0.792] |

*EI restricts positives to the 2,125 IUs agreed by both raters. AUC gain (+0.041) indicates label noise attenuates EU performance.*
