# Supplementary material for: Recognition of eating episodes via commercial smartwatch sensors analysis
Source: PLOS Digit Health. 2026 Jul 7;5(7):e0001539. doi: 10.1371/journal.pdig.0001539 (PMC13340811; doi:10.1371/journal.pdig.0001539)
Supplement: S6 Table — Same raw 5 Hz stream, 19 subjects, 26,058 IUs, 27.0% eating, same XGBoost-tuned hyperparameters. Panel A: marginal LOSO performance of each pipeline. Panel B: paired cluster-bootstrap comparison (filtered minus unfiltered). (DOCX) [file pdig.0001539.s007.docx]

## S6 Table. Filter-vs-no-filter ablation (Butterworth 0.3 Hz vs window-aggregation denoising).

Same raw 5 Hz stream, 19 subjects, 26,058 IUs, 27.0% eating. Same XGBoost-tuned hyperparameters.

### Panel A. Marginal performance (cluster-bootstrap 95% CI, B = 1000, seed = 1812)

| Pipeline | Sensitivity | Specificity | Balanced accuracy | AUC |
| --- | --- | --- | --- | --- |
| No filter (implicit denoising) | 0.605 [0.522, 0.684] | 0.689 [0.625, 0.750] | 0.647 [0.621, 0.674] | 0.710 [0.670, 0.750] |
| Butterworth 0.3 Hz | 0.572 [0.482, 0.656] | 0.706 [0.636, 0.775] | 0.639 [0.613, 0.667] | 0.703 [0.668, 0.738] |

### Panel B. Paired comparison (filtered − unfiltered)

| Metric | Δ | 95% CI | p |
| --- | --- | --- | --- |
| Sensitivity | **−0.033** | [−0.053, −0.016] | **0.002** |
| Specificity | +0.016 | [+0.002, +0.030] | 0.020 |
| Balanced accuracy | −0.008 | [−0.018, +0.001] | 0.088 |
| AUC | −0.007 | [−0.017, +0.004] | 0.216 |

*The filter significantly reduces sensitivity (p = 0.002) without improving balanced accuracy or AUC. The implicit feature-level denoising (§2.3) is preferable on 5 Hz data (Fuscà et al., 2019).*
