# Supplementary material for: Recognition of eating episodes via commercial smartwatch sensors analysis
Source: PLOS Digit Health. 2026 Jul 7;5(7):e0001539. doi: 10.1371/journal.pdig.0001539 (PMC13340811; doi:10.1371/journal.pdig.0001539)
Supplement: S1 Appendix — Fully nested subject-level cross-validation and the post-selection optimism gap (Table S1.1); full versus sensor-only feature-set ablation (Table S1.2); multiplicity-adjusted pairwise model comparisons across the four metrics (Table S1.3); inter-rater agreement at the Information-Unit and raw-frame levels, with the exact rater-by-rater contingency table and Cohen’s kappa (Table S1.4); and the meal-blocked few-shot personalisation experiment (Table S1.5). Also includes the companion-repository data-file inventory and references. (DOCX) [file pdig.0001539.s008.docx]

# S1 Appendix

Supplementary analyses for the Round-2 revision of *Recognition Of Eating Episodes Via Commercial Smartwatch Sensors Analysis* (PDIG-D-26-00312R1)

This appendix collects the additional analyses requested in the second round of review. They confirm the conclusions of the main text: under subject-independent (leave-one-subject-out) validation, tuned XGBoost remains the numerically best classifier on balanced accuracy and AUC, the performance is driven by wrist motion alone, and the residual error is dominated by inter-subject variability. Table S1.1 reports a fully nested subject-level cross-validation and the resulting optimism relative to the post-selection estimates of the main text. Table S1.2 reports the sensor-only ablation. Table S1.3 reports the multiplicity-adjusted pairwise model comparisons. Table S1.4 reports the inter-rater agreement with exact percent agreement and the exact Cohen's kappa computed from the two recovered rater streams. Table S1.5 reports the meal-blocked few-shot personalisation experiment. Unless stated otherwise, every point estimate is a mean across the 19 leave-one-subject-out subjects, reported with a subject-level (cluster) non-parametric bootstrap 95% confidence interval (B = 1,000, seed = 1812), in which the subject is the unit of resampling and therefore the unit of independent replication (N = 19). Confidence intervals are written as [lower, upper].

The Round-1 supporting information used the numbering S1 Fig and S1 to S7 Table; the Round-1 S5 Table is removed as superseded (see the Manuscript changes document). For clarity these new tables are presented here as a self-contained S2 Appendix numbered S2.1 to S2.5; folding them into a single continuous sequence with the retained Round-1 tables is trivial if the journal prefers it.

## Table S1.1. Nested versus post-selection leave-one-subject-out performance (optimism gap)

For each of the 19 outer held-out subjects, the tuned tree-model hyperparameters were selected using only the other 18 subjects, through an inner subject-grouped 5-fold cross-validation over the same Latin-hypercube search space and with the same selection rule (best mean sensitivity) as the main analysis, refit on those 18 subjects, and evaluated once on the held-out subject. The Transformer architecture was fixed a priori, so only its decision threshold was selected in the same nested way and then applied to the held-out subject; the pooled threshold over all 19 folds (0.29) was close to the value used in the main text (0.30). The column "Nested LOSO" reports the resulting estimates; the column "Original LOSO" reproduces the post-selection estimates of Tables 2 and 3; the last column is the nested balanced accuracy minus the original balanced accuracy. The nested and original estimates differ by at most 0.005 in balanced accuracy for the tuned tree models, and the nested estimates are marginally higher, so there is no evidence of post-selection optimism. For the Transformer, nesting the threshold leaves balanced accuracy and AUC essentially unchanged and lowers the sensitivity from 0.691 to 0.649, because the original threshold had been chosen on the pooled leave-one-subject-out predictions.

| Model (tuned) | Nested LOSO (sens, spec, bal. acc., AUC) | Original LOSO (sens, spec, bal. acc., AUC) | Balanced accuracy difference |
| --- | --- | --- | --- |
| XGBoost | 0.594 [0.515, 0.672], 0.691 [0.627, 0.747], 0.643 [0.612, 0.674], 0.712 [0.670, 0.752] | 0.593 [0.512, 0.677], 0.692 [0.624, 0.756], 0.642 [0.612, 0.671], 0.712 [0.671, 0.749] | higher by 0.001 |
| Random Forest | 0.244 [0.178, 0.314], 0.917 [0.883, 0.948], 0.580 [0.557, 0.605], 0.692 [0.649, 0.732] | 0.240 [0.174, 0.311], 0.915 [0.883, 0.948], 0.578 [0.554, 0.601], 0.691 [0.648, 0.728] | higher by 0.002 |
| LightGBM | 0.299 [0.229, 0.369], 0.885 [0.847, 0.921], 0.592 [0.568, 0.617], 0.694 [0.654, 0.733] | 0.281 [0.204, 0.369], 0.893 [0.852, 0.929], 0.587 [0.560, 0.614], 0.696 [0.652, 0.734] | higher by 0.005 |
| Transformer | 0.649 [0.584, 0.709], 0.531 [0.475, 0.586], 0.590 [0.566, 0.617], 0.646 [0.613, 0.678] | 0.691 [0.637, 0.735], 0.504 [0.456, 0.549], 0.597 [0.570, 0.624], 0.646 [0.613, 0.679] | lower by 0.007 |

The Transformer architecture had itself been chosen among six candidates on cross-validated performance, so a small residual selection element remains in that one choice; the six candidates differed by at most about 0.02 in balanced accuracy, which bounds how much that residual can matter.

## Table S1.2. Full versus sensor-only feature set

The sensor-only feature set comprises the 59 motion-derived predictors (7 slope coefficients of the raw signals against time, 49 right-wrist window statistics, and 3 axis cross-correlations). The full feature set adds the 16 experimental-context dummies (1 for study arm or menu, 3 for meal, and 12 for food) that an autonomous watch would not have at prediction time. The three tuned tree models were evaluated under the nested protocol of Table S1.1. Logistic Regression and the Decision Tree have no hyperparameter selection step, so they are valid final-test estimates under plain (un-nested) leave-one-subject-out, which is the appropriate design for models with no selection step; they were re-fit on each feature set with the same class weighting and the same subject-level bootstrap, and their full-feature point estimates reproduce the main-text Table 2. The reduction columns are the full estimate minus the sensor-only estimate. All reductions are small and fall within overlapping confidence intervals; among the tuned tree models the largest is the LightGBM AUC (lower by 0.016), and among the baselines the largest is the Logistic Regression AUC (lower by 0.021). The sensor-only XGBoost remains the numerically best classifier on both balanced accuracy and AUC across all five models. The paired statistical comparison of Logistic Regression with XGBoost on balanced accuracy is reported on the full feature set in Table S1.3 (difference 0.009, adjusted p = 0.435); for the sensor-only feature set we report point estimates and confidence intervals only, without a paired test, and the sensor-only point difference between the two is 0.019. The menu sub-group analysis in the main text (Section 3.6) does not substitute for this ablation, because it does not remove the food and meal variables from the feature matrix.

| Model | Feature set | Sensitivity | Specificity | Balanced accuracy | AUC |
| --- | --- | --- | --- | --- | --- |
| XGBoost (tuned) | Full (75) | 0.594 [0.515, 0.672] | 0.691 [0.627, 0.747] | 0.643 [0.612, 0.674] | 0.712 [0.670, 0.752] |
| XGBoost (tuned) | Sensor-only (59) | 0.594 [0.528, 0.663] | 0.683 [0.630, 0.732] | 0.639 [0.607, 0.668] | 0.699 [0.653, 0.743] |
| Random Forest (tuned) | Full (75) | 0.244 [0.178, 0.314] | 0.917 [0.883, 0.948] | 0.580 [0.557, 0.605] | 0.692 [0.649, 0.732] |
| Random Forest (tuned) | Sensor-only (59) | 0.226 [0.171, 0.283] | 0.928 [0.904, 0.952] | 0.577 [0.555, 0.601] | 0.685 [0.642, 0.725] |
| LightGBM (tuned) | Full (75) | 0.299 [0.229, 0.369] | 0.885 [0.847, 0.921] | 0.592 [0.568, 0.617] | 0.694 [0.654, 0.733] |
| LightGBM (tuned) | Sensor-only (59) | 0.265 [0.214, 0.319] | 0.894 [0.866, 0.923] | 0.580 [0.556, 0.603] | 0.678 [0.637, 0.720] |
| Logistic Regression | Full (75) | 0.625 [0.548, 0.704] | 0.643 [0.585, 0.698] | 0.634 [0.606, 0.658] | 0.697 [0.662, 0.729] |
| Logistic Regression | Sensor-only (59) | 0.597 [0.510, 0.683] | 0.642 [0.575, 0.706] | 0.620 [0.589, 0.652] | 0.676 [0.636, 0.716] |
| Decision Tree | Full (75) | 0.572 [0.484, 0.653] | 0.659 [0.594, 0.731] | 0.616 [0.588, 0.641] | 0.649 [0.616, 0.683] |
| Decision Tree | Sensor-only (59) | 0.536 [0.449, 0.608] | 0.690 [0.626, 0.758] | 0.613 [0.584, 0.639] | 0.639 [0.606, 0.670] |

Reduction in balanced accuracy and AUC (full minus sensor-only): XGBoost, lower by 0.004 and lower by 0.013; Random Forest, lower by 0.003 and lower by 0.007; LightGBM, lower by 0.012 and lower by 0.016; Logistic Regression, lower by 0.014 and lower by 0.021; Decision Tree, lower by 0.003 and lower by 0.010.

## Table S1.3. Multiplicity-adjusted pairwise comparisons against tuned XGBoost

Paired subject-level cluster bootstrap (B = 1,000, seed = 1812) on the final per-subject metrics (the nested tree models, the Transformer with the nested threshold, and the default Attention model), with p-values adjusted across the whole family of comparisons using the Benjamini and Hochberg false-discovery-rate procedure and the more conservative Holm procedure. The family contains 180 comparisons (45 pairs across the 4 metrics among 10 models, namely the eleven classifiers of Tables 2 and 3 excluding the tuned Attention configuration, which is reported there for completeness but is represented in this comparison by the default Attention model); the complete matrix is the backing dataset for this table. Of the 180 comparisons, 136 are significant at raw p below 0.05, 133 remain significant after the false-discovery-rate adjustment, and 111 after Holm, so the adjustment changes the overall picture only slightly. Each "Difference" entry is the comparator relative to tuned XGBoost. Probabilities below 0.001 are shown as such.

### Balanced accuracy

| Comparator | Difference versus tuned XGBoost | Raw p | Benjamini-Hochberg p | Holm p |
| --- | --- | --- | --- | --- |
| Logistic Regression | lower by 0.009 | 0.372 | 0.435 | 1.00 |
| Decision Tree | lower by 0.027 | 0.006 | 0.009 | 0.360 |
| XGBoost (default) | lower by 0.028 | <0.001 | <0.001 | <0.001 |
| Attention pooling (default) | lower by 0.033 | <0.001 | <0.001 | <0.001 |
| LightGBM (tuned) | lower by 0.050 | <0.001 | <0.001 | <0.001 |
| Transformer | lower by 0.053 | <0.001 | <0.001 | <0.001 |
| LightGBM (default) | lower by 0.053 | <0.001 | <0.001 | <0.001 |
| Random Forest (tuned) | lower by 0.063 | <0.001 | <0.001 | <0.001 |
| Random Forest (default) | lower by 0.065 | <0.001 | <0.001 | <0.001 |

### Sensitivity

| Comparator | Difference versus tuned XGBoost | Raw p | Benjamini-Hochberg p | Holm p |
| --- | --- | --- | --- | --- |
| Transformer | higher by 0.055 | 0.144 | 0.180 | 1.00 |
| Logistic Regression | higher by 0.031 | 0.100 | 0.128 | 1.00 |
| Decision Tree | lower by 0.022 | 0.526 | 0.581 | 1.00 |
| Attention pooling (default) | lower by 0.073 | 0.004 | 0.006 | 0.256 |
| XGBoost (default) | lower by 0.178 | <0.001 | <0.001 | <0.001 |
| LightGBM (tuned) | lower by 0.295 | <0.001 | <0.001 | <0.001 |
| LightGBM (default) | lower by 0.299 | <0.001 | <0.001 | <0.001 |
| Random Forest (tuned) | lower by 0.351 | <0.001 | <0.001 | <0.001 |
| Random Forest (default) | lower by 0.371 | <0.001 | <0.001 | <0.001 |

### Specificity

| Comparator | Difference versus tuned XGBoost | Raw p | Benjamini-Hochberg p | Holm p |
| --- | --- | --- | --- | --- |
| Attention pooling (default) | higher by 0.007 | 0.820 | 0.853 | 1.00 |
| Decision Tree | lower by 0.032 | 0.294 | 0.355 | 1.00 |
| Logistic Regression | lower by 0.049 | 0.034 | 0.046 | 1.00 |
| XGBoost (default) | higher by 0.123 | <0.001 | <0.001 | <0.001 |
| Transformer | lower by 0.160 | <0.001 | <0.001 | <0.001 |
| LightGBM (default) | higher by 0.193 | <0.001 | <0.001 | <0.001 |
| LightGBM (tuned) | higher by 0.194 | <0.001 | <0.001 | <0.001 |
| Random Forest (tuned) | higher by 0.226 | <0.001 | <0.001 | <0.001 |
| Random Forest (default) | higher by 0.240 | <0.001 | <0.001 | <0.001 |

### AUC

| Comparator | Difference versus tuned XGBoost | Raw p | Benjamini-Hochberg p | Holm p |
| --- | --- | --- | --- | --- |
| Random Forest (default) | lower by 0.012 | 0.010 | 0.014 | 0.550 |
| Logistic Regression | lower by 0.014 | 0.244 | 0.299 | 1.00 |
| LightGBM (tuned) | lower by 0.018 | <0.001 | <0.001 | <0.001 |
| XGBoost (default) | lower by 0.019 | <0.001 | <0.001 | <0.001 |
| Random Forest (tuned) | lower by 0.020 | <0.001 | <0.001 | <0.001 |
| LightGBM (default) | lower by 0.021 | <0.001 | <0.001 | <0.001 |
| Decision Tree | lower by 0.063 | <0.001 | <0.001 | <0.001 |
| Transformer | lower by 0.066 | <0.001 | <0.001 | <0.001 |
| Attention pooling (default) | lower by 0.069 | <0.001 | <0.001 | <0.001 |

On balanced accuracy, only Logistic Regression is statistically indistinguishable from tuned XGBoost (lower by 0.009, adjusted p = 0.435); the Decision Tree is borderline (lower by 0.027, significant under the false-discovery-rate adjustment but not under Holm); every other model is lower and survives both adjustments. The higher sensitivity of the Transformer relative to tuned XGBoost (higher by 0.055) has a 95% confidence interval that runs from slightly below zero to about 0.12 and is not significant (raw p = 0.144, adjusted p = 0.18), while its specificity is lower by 0.160 (95% confidence interval 0.10 to 0.22, p < 0.001). With only 19 subjects the subject-level bootstrap p-values are themselves approximate, so the interpretation in the main text rests on effect sizes and confidence intervals rather than on the survival counts above.

## Table S1.4. Inter-rater agreement at the Information Unit and raw-frame levels

The two evaluators annotated the eating bites independently, and the two label streams are retained separately in the labelling pipeline that produced the analysis datasets. We reconstructed each evaluator's eating indicator at the 5 Hz frame rate using the project's own labelling function, and we verified the reconstruction exactly: the union of the two streams reproduces the stored eating-union label with no discrepancy at the frame level, and aggregating the streams into Information Units reproduces the published Information Unit dataset exactly (26,304 units, with identical eating-union and eating-intersect prevalences). The percent agreement does not depend on how the contested cases are divided between the two raters and is exact; with the two streams available, Cohen's kappa is identified exactly as well. The actual rater-by-rater contingency table and the exact kappa follow.

| Level | N | Both eating | Rater 1 only | Rater 2 only | Both non-eating | Percent agreement | Cohen's kappa | Landis and Koch band |
| --- | --- | --- | --- | --- | --- | --- | --- | --- |
| Information Unit (window length 5 s) | 26,304 | 2,286 | 4,411 | 284 | 19,323 | 82.2% | 0.41 | moderate |
| Raw frame (5 Hz) | 133,040 | 22,636 | 15,622 | 1,283 | 93,499 | 87.3% | 0.65 | substantial |

Direction of the disagreement. The two streams differ systematically, and that direction is precisely what an even-split reconstruction cannot recover. At the Information Unit level the disagreement is almost entirely one-sided: of the 4,695 contested Information Units, 4,411 (94%) were labelled eating by the first rater alone and only 284 by the second alone. The first rater labelled eating in 25.5% of Information Units against 9.8% for the second, so the pooled eating-union label tracks the first rater's stream and the eating-intersect label tracks the second. At the raw-frame level the relationship is the same and the agreement is higher (Cohen's kappa 0.65, substantial). The lower Information Unit kappa (0.41, moderate) reflects the stricter eating prevalence after majority-vote windowing, together with the concentration of the residual disagreement at the boundaries of eating episodes, rather than poor labelling. Agreement and kappa vary across participants (Information Unit agreement from 44% to 97%; per-subject kappa from 0.09 to 0.86), with the full per-subject values in the companion repository.

## Table S1.5. Meal-blocked few-shot personalisation of tuned XGBoost

Calibration examples (eating units only) were drawn from each subject's first two meals, and performance was evaluated only on that subject's remaining two meals, so that calibration and test windows come from separate recording sessions and cannot overlap. The baseline and the personalised models were evaluated on the same held-out meal block, and the sensor-only feature set was used. The five lower-performing subjects (difficult group: 08, 09, 10, 11, 16) were the bottom five by baseline leave-one-subject-out sensitivity under default-hyperparameter XGBoost; the remaining 14 form the other group. Mean baseline sensitivity, mean few-shot sensitivity, and the mean change are reported by group and by number of calibration examples. At the largest setting (100 examples) a few subjects had fewer than 100 eating Information Units available in their first two meals (for example, subject 16 had 88), in which case all available units were used. Values are rounded independently to three decimals; the change column is the difference of the unrounded means.

| Group (subjects) | Calibration examples | Mean baseline sensitivity | Mean few-shot sensitivity | Mean change |
| --- | --- | --- | --- | --- |
| Difficult (5) | 5 | 0.278 | 0.309 | higher by 0.031 |
| Difficult (5) | 10 | 0.278 | 0.338 | higher by 0.059 |
| Difficult (5) | 20 | 0.278 | 0.292 | higher by 0.014 |
| Difficult (5) | 50 | 0.278 | 0.328 | higher by 0.050 |
| Difficult (5) | 100 | 0.278 | 0.328 | higher by 0.050 |
| Other (14) | 5 | 0.399 | 0.397 | lower by 0.002 |
| Other (14) | 10 | 0.399 | 0.413 | higher by 0.015 |
| Other (14) | 20 | 0.399 | 0.418 | higher by 0.019 |
| Other (14) | 50 | 0.399 | 0.436 | higher by 0.037 |
| Other (14) | 100 | 0.399 | 0.422 | higher by 0.023 |

On the difficult group the change was modest, non-monotonic, and noisy, ranging from higher by 0.014 to higher by 0.059 across the different numbers of calibration examples; at 100 examples it was higher by 0.050. On the other group the change was small, up to higher by 0.037, and was higher by 0.023 at 100 examples. The effect on the difficult group was driven mainly by a single participant, and the per-subject test sets were small, so the individual estimates are correspondingly uncertain.

Per-subject values at 100 calibration examples (difficult group):

| Subject | Test eating units | Baseline sensitivity | Few-shot sensitivity |
| --- | --- | --- | --- |
| 08 | 85 | 0.118 | 0.188 |
| 09 | 134 | 0.172 | 0.313 |
| 10 | 166 | 0.361 | 0.386 |
| 11 | 81 | 0.272 | 0.284 |
| 16 | 32 | 0.469 | 0.469 |

# Companion Repository Data Files

The following machine-readable data files are deposited in the companion repository (lucavd/recognition_of_eating_episodes, archived via Zenodo):

**Per-subject metrics**

| File | Description |
| --- | --- |
| per_subject_metrics.csv | Per-subject sensitivity, specificity, bal. accuracy, AUC for RF/XGB/LGB (default + tuned) |
| per_subject_lr_dt.csv | Per-subject metrics for Logistic Regression and Decision Tree |
| per_subject_lgb_tuned.csv | Per-subject metrics for LightGBM tuned |
| per_subject_attention_default.csv | Per-subject metrics for Attention pooling (default) |
| deep_per_subject.csv | Per-subject metrics for Attention tuned + Transformer |
| per_subject_xgb_tuned_EI.csv | Per-subject metrics for XGBoost tuned on EI target |
| per_subject_filter.csv | Per-subject metrics for filter-vs-no-filter ablation |

**Bootstrap and statistical outputs**

| File | Description |
| --- | --- |
| bootstrap_ci.csv | Cluster-bootstrap 95% CIs for all 11 classifiers |
| pairwise_nested_adjusted.csv | Multiplicity-adjusted pairwise comparison matrix: 45 model pairs × 4 metrics among the 10 final-table models, with raw, Benjamini-Hochberg and Holm p-values (backs S1 Appendix Table S1.3) |
| xgb_tuned_EI_ci.csv | Bootstrap CIs for XGBoost tuned on EI target |
| filter_ablation_ci.csv | Bootstrap CIs for filter-vs-no-filter ablation |
| filter_ablation_paired.csv | Paired comparison filtered vs unfiltered |
| mde.csv | Minimum detectable effect table (N = 19 to N = 200) |
| timeframe_ci.csv | Bootstrap CIs for window-size sensitivity (δs = 1–5) |

**Labelling and agreement**

| File | Description |
| --- | --- |
| per_subject_agreement.csv | Per-subject percent agreement and Cohen's kappa |
| inter_rater_exact.csv | Inter-rater agreement at the IU and raw-frame levels: 2 × 2 counts, percent agreement and exact Cohen’s kappa |

**Sub-analyses**

| File | Description |
| --- | --- |
| subgroup_arm.csv | Menu A vs Menu B stratified LOSO metrics |
| subgroup_arm_diff.csv | Between-menu bootstrap differences |
| fp_pattern_per_subject.csv | Per-subject false-positive pattern (boundary vs isolated) |
| inference_latency.csv | XGBoost inference latency benchmark |
| fewshot_blocked_per_subject.csv | Meal-blocked few-shot personalisation, per-subject results (group summary in fewshot_blocked_summary.csv) |

**Feature engineering**

| File | Description |
| --- | --- |
| feature_list.csv | Complete list of 75 predictor names |
| iu_features_unfiltered.rds | IU feature matrix, no upstream filter (26,058 IUs) |
| iu_features_filtered.rds | IU feature matrix, Butterworth 0.3 Hz (26,058 IUs) |

## References

Fuscà E, Bolzon A, Buratin A, Ruffolo M, Berchialla P, Gregori D, et al. Measuring Caloric Intake at the Population Level (NOTION): Protocol for an Experimental Study. JMIR Research Protocols. 2019;8: e12116. doi:10.2196/12116
